# Generating bat primary and immortalised cell-lines from wing biopsies

**DOI:** 10.1038/s41598-024-76790-3

**Published:** 2024-11-12

**Authors:** Dominic Alcock, Sarahjane Power, Bridget Hogg, Carlotta Sacchi, Joanna Kacprzyk, Sarah McLoughlin, Mads Frost Bertelsen, Nicola F. Fletcher, Aidan O’Riain, Emma C. Teeling

**Affiliations:** 1 UCD School of Biology and Environmental Science, University College Dublin, Dublin, Ireland; 2 UCD School of Veterinary Medicine, Veterinary Science Centre Belfield, University College Dublin, Dublin, Ireland; 3 Center for Zoo and Wild Animal Health, Copenhagen Zoo, Frederiksberg, Denmark; 4 UCD School of Veterinary Medicine, UCD Conway Institute, University College Dublin, Dublin, Ireland

**Keywords:** Chiroptera, Primary, Immortalisation, Cell culture, Biopsy, Cell line, Fibroblasts, SV40LT, Ageing, Wing punch, Non-lethal, Oxidative stress tolerance, Biological techniques, Cell biology, Immunology, Molecular biology, Zoology

## Abstract

Bats are becoming recognised as new model species to study naturally evolved mammalian extended healthspan and disease tolerance. However, this research is limited by the lack of bat specific cellular resources. Here we describe an optimised protocol to develop both primary and immortalised fibroblast cell-lines from wing biopsy punches from the Egyptian fruit bat, *Rousettus aegyptiacus*. We show that the immortalised cell lines and primary cells show similar characteristics in their proliferative capacity and response to oxidative stress. They also exhibited a similar response in their NF-κB immune response to TLR agonists including SARS-CoV2. As wing punches can be acquired non-lethally, these methods can be used to develop primary and immortalised cells, from potentially any bat species, including those of conservation concern that cannot be sacrificed. This can expand the scope of bat species that can be studied in the future, and the development of key cellular resources required to functionally validate the regulators of bats’ unique longevity.

**Supplementary Information:**

The online version contains supplementary material available at 10.1038/s41598-024-76790-3.

## Introduction

Bats are exceptional among mammals. All bats are placed into the Order Chiroptera accounting for 20% of mammals with ~ 1482 bat species found on every continent except Antarctica^1^. Bats occupy a broad range of ecological niches, feeding on arthropods, nectar, fruit, fish, small vertebrates, and blood^2^. Their global success has been attributed to the evolution of many unique mammalian traits. Most bats can use laryngeal echolocation, allowing them to orient with sound, detecting obstacles in flight and foraging for food in complete darkness^2^. With their forelimbs adapted as wings, they are the only mammals capable of true, self-powered flight—a highly metabolically costly trait. High metabolic rates have long been associated with small, short-lived mammals, yet bats have the longest recorded lifespan relative to body size of all mammals, on average living approximately 3.5 times longer than mammals of similar size and showing little signs of ageing^3,4^. Bats also are shown to asymptomatically host a diversity of viruses, including SARS-like-COVs, MERs-like-COVs, Hendra virus, Nipah virus, potentially due to a unique and tolerant immune response^5,6^. Given these distinctive traits, bats are now being considered as novel model species for studying the molecular basis of both extended healthspan^7–9^ and disease tolerance in mammals^10–12^.

Comparative analyses of bat genomes have uncovered multiple immune and longevity adaptations—including the diverse selection, expansion, and contraction of specific innate immune genes^10,13,14^ as well as selection across telomere maintenance^7^, DNA repair^15^, metabolic^16^ and proteostasis pathways^9^. However, the majority of the proposed bat molecular adaptations have yet to be functionally validated in the laboratory, stemming in part from limited bat specific cellular resources^11,12^, a limitation typical for non-model organisms.

As most bat species are wild, endangered, protected, and do not survive well in captivity, lethal sampling is typically prohibited, which limits the sample type and of tissue volume that can be taken^11^. To date, multiple primary and immortalised cultures from bats have been established; however the majority have been derived from lethally sampled organ tissues (reviewed in supplementary Table 1). Kacprzyk et al. (2016) was one of the first studies to establish primary fibroblasts derived from 3 mm wing membrane biopsies^17^ non-lethally sampled from wild lesser horseshoe bats (*Rhinolophus hipposideros*) and this protocol was further adapted to work across multiple species (e.g. *Myotis myotis*, *Pipistrellus kuhlii*, *Phyllostomus discolor*)^8,9,18^. However, it is well documented that primary cell cultures have a limited life span and cannot be passaged indefinitely. After a number of population doublings known as the ‘Hayflick limit’^19^ - determined by species, cell type, and culture conditions—primary cells enter a state of replicative senescence, wherein cells cease to further divide^20^. Hence, while passaging can be used to prolong the use of primary cell cultures, it can only do so for a finite number of times, thus limiting the amount of material available for functional assays. Alternatively, immortalised cell lines, comprised of a single cell type that can be serially propagated, and can be generated via a number of methods including transduction, transfection, and overexpression of telomerase^21–23^. In the context of functional studies, primary cultures are often preferred. While immortalised cell lines can provide a limitless resource, their transformation and continuous passaging may affect their responses and could change their in-vivo characteristics. Primary cells are therefore considered a more representative model of the in-vivo state.

Despite these limitations, experiments using cultured bat cells have further identified and validated unique bat molecular adaptations, such as the discovery of genotoxic protection conferred by the enhanced ABCB1 transporter system using *Pteropus alecto* cell cultures, both primary and immortalised, from a number of cell tissue origins^24^. Cultured bat cells have also been used to identify novel viruses such as RV1 from *Pteropus dasymallus*^25^ and two adenoviruses originating from *Myotis macrodactylus*^26^, and have shown how bats may tolerate these viruses through altered NLRP3 inflammasome activation in *Pteropus alecto*^27^, confirming genomic predictions. Bat organoid models were used to show a lack of viral amplification in *Rousettus leschenaultii* when infected with SARS-CoV-2^28^. Aspects of the ageing process have also been investigated within bat cells revealing that bats exhibit elevated autophagy compared with shorter lived species and that this increases with age^9,29^. Furthermore bat cells have been shown to be more resistant to a multitude of toxic stressors such as rotenone and hydrogen peroxide^30^, compared with other mammals. These results highlight the utility and need to generate bat cellular resources, from multiple bat species, in order to fully develop bats as a new model study species of disease resistance and extended healthspan^11^.

Here we developed an optimised protocol for the establishment of primary and immortalised cell cultures for bats that can be generated from a non-lethal sample type, a wing punch biopsy. We used wing biopsies taken from captive *Rousettus aegyptiacus*, an ideal candidate species to establish such protocols due to its wide availability in zoos and labs, non-threatened conservation status, and potential as a model study species for disease tolerance. We employed the SV40 large T antigen (SV40LT) to immortalise the generated primary fibroblasts and compared the response of primary and immortalised cells to immunological and cellular stressors, with both cell types showing a similar response. These methods could be potentially used for any bat species, providing the enhanced conditions and considerations required to grow primary cell cultures from non-lethally sampled wing punches and the development of an immortalised permanent cell line for downstream manipulation. These steps are required to establish bats as new model study species by developing the cellular resources required to functionally validate the genomic predictions of the regulators that underlie bats’ unique adaptations.

## Methods

### Generation of primary cultures

#### Sample collection

Wing tissues were obtained from captive female Egyptian fruit bats (*Rousettus aegyptiacus*) from the Copenhagen Zoo, Denmark following euthanasia by trained veterinarians in line with management decisions in the zoo to ensure best welfare of the captive colony^31^. All individuals were aged under 12 months old. Euthanised bats were manoeuvred into a supine position and with the wing manually extended, multiple biopsies were taken from the plagiopatagium of each individual using sterile 4 mm wing biopsy punches. This enabled biological replicates from the same individual, which is typically not possible with wild bats. Single 3–4 mm biopsy punches are typically sampled from live bats per wing and have been shown to heal quickly with no adverse effects^32,33^. Biopsies were immediately transferred to cryogenic tubes (Sarstedt: 72.379) containing fresh culture medium composed of Dulbecco’s modified eagle’s medium (DMEM) (GIBCO: 41966029) supplemented with 20% foetal bovine serum (FBS) (GIBCO: 10500064), and 1% antibiotic-antimycotic (Gibco: 15240096). Biopsies were stored at 4 °C for transport until such a time that they could be frozen, at which point they were then transferred to a solution of 90% FBS and 10% dimethyl sulfoxide (DMSO) (Thermofisher: D8418) and stored at − 80 °C using a Mr.Frosty™ (Thermofisher: 5100–0001).

#### Enhanced digestion media setup for cell extraction assessment

Frozen biopsies (*n* = 3 biopsies from 3 individuals, Supplementary Table 2) were removed from the − 80 °C freezer and immediately transferred to a 37 °C water bath to rapidly thaw. With a small ice particle still remaining, the vial was quickly moved from the water bath into the laminar flow hood for processing. The freezing solution was gently removed, and biopsies were left to soak in medium with high concentration of antibiotic (3%) for 20 min in order to minimise the risk of contamination. To increase digestion surface area, tissues were minced using a sterile blade (Swann-Morton: 0508) before being placed into the digestion solution.

Following this, three digestion protocols were evaluated to maximise the number of cells released from tissues and accelerate cell proliferation post-digestion. All protocols included collagenase type II (Col 2) (Thermofisher: 17101015) that was used previously^17^ with post digestion centrifugation to maximise cell numbers as evidenced in a pilot study^34^. As elastin has been suggested to maintain tissue integrity and structure in the bat wing membrane^35,36^, elastase was included in two variations of the protocol, aimed to enhance yield of released cells. The digestion recipes used here constituted 500 µl of either (1) DMEM + 20% FBS + 0.1% Col 2; (2) DMEM + 20% FBS + 0.1% Col 2 + 0.04% Elastase (Merck: E1250-10MG); and, (3) Fibroblast Growth Media (FGM) (ATCC: PCS-201-041) + 20% FBS + 0.1% Col 2 + 0.04% Elastase. Tissue was digested overnight (18 h) in a 2 ml cryovial with lid slightly loosened to promote gaseous exchange within an incubator set at 37 °C, 5% CO_2_.

After digestion, the digest was mixed with 2 ml of the culture medium and plated on 35 mm sterile cell culture plates (Sigma-Aldrich: 10556661). These were then left undisturbed at standard growth conditions (37 °C and 5% CO_2_). The cells were fed every three days with prewarmed fresh culture medium. When initial attachment of fibroblasts occurred in plates and it was clear that no bacterial or fungal contamination was present, antibiotic concentration was lowered to 0.2%.

#### Digestion media comparisons

The cells were harvested when the first plate per experimental repeat reached 80–90% confluence, which typically occurred after 8 days. After removal of plates from the incubator, the medium was aspirated and 800 µL of prewarmed 0.05% Trypsin-EDTA (Ethylenediaminetetraacetic acid) (Gibco: 25300054) was added to each plate. Plates were incubated for 5 min before the trypsin was neutralised by adding fresh prewarmed culture medium at a ratio of 3:1 medium to trypsin. The solution was gently pipetted up and down to dislodge any still attached cells, and then transferred to a falcon tube and spun at 300 g for 5 min. The cells were then resuspended in 1 ml of media and counted using toluidine blue staining on a haemocytometer.

A Mann–Whitney U test with FDR corrections (p-value of 0.05) was carried out in R^37^ to determine if there was a statistical difference in cell count between protocols.

##  Immortalisation of primary cultures

### Resuscitation of primary cells

Frozen vials containing 1 × 10^6^/ml *Rousettus* primary cells from individuals R42, R46 and R49 derived from the enhanced FGM + Col 2 + Elastase formulation, were thawed quickly to avoid ice crystal formation. A small amount of DMEM complete: DMEM, 20% FBS, and 1% Minimal Essential Medium Non-Essential Amino Acids (NEAA) (GIBCO: 11140068) was added to the vial to start the dilution of the freezing media to not shock the cells with a rapid change in cryopreservant concentration. The cells were then added to a final volume of 9 ml of DMEM complete and spun for 5 min at 300 g. The pellet was resuspended gently in 1 ml of DMEM complete and added to 3 ml of DMEM complete in a T25 flask (Sigma-Aldrich: C6481-200EA). The cells were passaged when they each reached 70–90% confluency. The cells were fed fresh media every three to four days.

### Passaging

Spent media was discarded and the cells were washed with Dulbecco’s Phosphate Buffered Saline (DPBS) (Biowest: L0615) (10 ml for a T75 and 3 ml for a T25). 0.05% Trypsin-EDTA (GIBCO: 25300054) (3 ml for a T75 and 1 ml for a T25) was then added to the surface of the cells. The flasks were incubated at 37 °C until the cell layer had lifted and the cells dissociated, approximately 5–10 min. The trypsin was then neutralised by the addition of media at 3:1 ratio. Following this the cells were centrifuged at 300 g for 5 min and counted manually using a haemocytometer. T75 (Sigma-Aldrich: C7231-120EA) flasks were seeded at a density of 1 × 10^6^ in 15 ml of media.

###  Transformation using SV40LT

High Titer Lentivirus expressing SV40LT(10^9^ IU/ml) from (ABM: LV613) that confers Puromycin resistance was used to immortalise the primary cell cultures R42, R46 and R49. To reduce the possibility of somatic drift the primary cell cultures were immortalised within four passages of initial establishment. Before carrying out the immortalisation a “kill curve” from 0.5 µg to 6 µg/ml of puromycin (GIBCO: A1113803) was performed to determine the lowest dose of puromycin that kills all cells, which through this approach was determined to be 0.5 µg/ml. The cells were transduced using the protocol described in the supplementary text (S1).

###  SV40LT staining

Cells were grown on circular coverslips for 48 h to allow attachment and then fixed in 100% ice cold methanol (Sigma-Aldrich: 646377) for 20 min. Following fixing, the coverslips were washed with Phospho Buffered Saline (PBS) (Gibco: 10010023) and stored at 4 °C until staining. Cells were probed with the Anti-SV40LT primary antibody [PAb416] (Abcam: ab16879) (1 in 200) and the Goat Anti-Mouse IgG H&L (Alexa Fluor^®^ 488) secondary antibody (Abcam: ab150113) (1 in 500). They were stained according to the manufacturer’s instructions and the nucleus was counterstained with DAPI (Thermofisher: 62248). Staining was then visualised on an epifluorescent microscope with filters corresponding to either DAPI or GFP for the Alexa Fluor^®^ 488 (Supplementary Fig. 1).

## Immortalised versus primary cells comparisons—cellular proliferation, maximum passage number, H2O2 stress and TLR response

###  Cellular proliferation

We compared two immortalised cell lines R42IM and R49IM (multiplicity of infection 10) with two primary cell cultures R42 and R49 (passage 10) generated from wing punches of the same individuals as detailed above to ascertain any differences in their cellular dynamics. The cells were seeded in 96 well plates (Corning: 3595) with 10,000/well and left for 24 h in a 37 °C incubator with 5% CO_2_ in 100 µl of complete DMEM medium containing (DMEM + 20% FBS + 1% NEAA) to allow the attachment of the cells to the well surface. After incubation the remaining media was aspirated and replaced with serum-free DMEM media to halt cell cycle progression (condition 1), and DMEM complete to allow normal cell growth (condition 2), for each culture (R42, R49, R42IM, R49IM). Six technical repeats were performed including three no cell control wells per media type. After 48 h of incubation (37 °C, 5% CO_2_), 10 µl of MTT (3-(4,5-dimethylthiazol-2-yl)-2,5-diphenyltetrazolium bromide) (5 mg/ml) reagent was added (Merck: 475989), and the cells were incubated for three more hours before removal of all media. 100 µl of DMSO (Sigma-Aldrich: D8418) was then added to solubilise the crystals, pipetting up and down briefly. Cellular proliferation was measured via an MTT assay, which measures the activity of mitochondrial reductases, recording absorbance at 590 nm in a plate reader (SPECTROstar^NANO^). The final absorbance was corrected for each well through subtraction of the average no cell control well absorbance. This overall procedure was repeated three times for each cell culture.

Corrected absorbance scores per technical repeats were averaged per biological replicate and used to assess doubling times using the following equation [$\:DT\:=\:48*\left(log\right(2)/(log\left(N\right)-log\left(SS\right)\left)\right)$, DT = Doubling Time, N = MTT value absorbance value of normal growth media cells; SS = MTT value absorbance value of serum starved cells] as described previously^38^. Statistical differences between immortalised versus primary cell proliferation were estimated using two tailed t-tests using Microsoft Excel.

### Maximum passage number

To identify the passage number at which primary fibroblasts and immortalised cell lines reach the replicative senescence, R42, R49, R42IM, R49IM cells (one T25 flask each) were cultured continuously in DMEM complete with media changes every 3–4 days or passaging whichever was required first in a 37 °C, 5% CO_2_ incubator. Cells were passaged when confluency reached 80% and reseeded in a 1:4 split, with this repeated until the cultures could no longer reach confluency after seeding and displayed the morphological properties of an enlarged and flattened surface, indicative of replicative senescence.

### Oxidative stress assay

Using the same cell cultures described above (R42, R49, R42IM, R49IM), we performed an oxidative stress test to compare the response of immortalised and primary cells derived from the same individuals. As before the cells were seeded in 96 well plates (Corning: 3595) with 10,000 cells/ well and left for 24 h in a 37 °C incubator with 5% CO_2_ in 100 µl of complete DMEM medium containing (DMEM + 20% FBS + 1% NEAA) to allow the attachment of the cells to the well surface. After this time all media was removed and replaced with a range of concentrations of H_2_O_2_ (Sigma-Aldrich: 88597) from 0 µM to 2000 µM prepared in DMEM complete and incubated for 24 h (37 °C, 5% CO_2_). Three technical repeats were performed for each cell culture, including three no cell controls containing media only. After this H_2_O_2_ exposure, wells were aspirated removing all media and 100 µl of DMEM and 10 µl of MTT (5 mg/ml) was added allowing for 3 h of incubation in the incubator at 37 °C, 5% CO_2_. Cell absorbance was measured and corrected as detailed above. This overall procedure was repeated three times. The percentage cell viability per H_2_O_2_ concentration was calculated relative to the 0 µM unexposed cells which was considered as 100% viability. An ANOVA permutation test with 5000 permutations was used to test for significance differences in oxidative stress response dependent on the cell line with an empirical p-value of 0.05 using R. The point at which cell viability was significantly affected by H_2_O_2_ concentration was also determined through ANOVA testing within R using Tukey corrections. The LD50 values for each cell culture repeat was calculated using the drc package^39^ within R and analysed through ANOVA for any differences in this value between all cell cultures.

### TLR agonist assay

Response of primary and immortalised bat cell cultures to toll-like receptor (TLR) agonists, and heat inactivated SARS-CoV-2, was carried out using an NF-κB luciferase reporter assay as previously described^40^ for primary cell cultures R42 and R49 and their immortalised counterparts (R42IM, R49IM). Two wells of a six well plate were seeded with either wild-type cells or the corresponding immortalised cell cultures at 5 × 10^5^ cells/well in 4 ml of complete DMEM and incubated at 37^o^C, 5% CO_2_ for 24 h. After 24 h, 7.5 µg of the NF-κB luciferase reporter plasmid was transfected into target cells using ViaFect^Tm^ transfection reagent in serum free DMEM. Cells were incubated at 37^o^C, 5% CO_2_ for 24 h. Cells were treated with TLR agonists from the Human TLR1-9 Agonist kit (Invivogen: tlrl-kit1hw) at 100 ng/ml unless stated otherwise in the manufacturer’s instructions and or SARS-CoV-2 (strain nCov-INMI-1 heat inactivated at 60^o^C for 30 min). Following incubation at 37^o^C, 5% CO_2_ for 24 h cells were lysed with passive lysis buffer and assayed for luciferase activity using the Promega Luciferase Assay Kit (Promega: E1500). The determination of differences in the TLR agonist assay using statistical analyses were performed using the Student t-test in Prism 9.0 (GraphPad) with a p-value of 0.05 being considered statistically significant.

##  Species identification confirmation

The species identity (*R.aegyptiacus)* of all primary and immortalised cultures was performed through the PCR amplification of mitochondrial Cytochrome b using primers designed previously^41^ with subsequent Sanger sequencing and verification of species using a BLAST search.

Primers used:

Forward: mtDNA-R3-F 5′-TGGCATGAAAAATCACCGTTGT-3′.

Reverse: mtDNA-F3-R 5′-AGGATGGCGTATGCAAATAGGAA-3′.

## Results

### Primary fibroblast culture optimisation

**Fig. 1:**
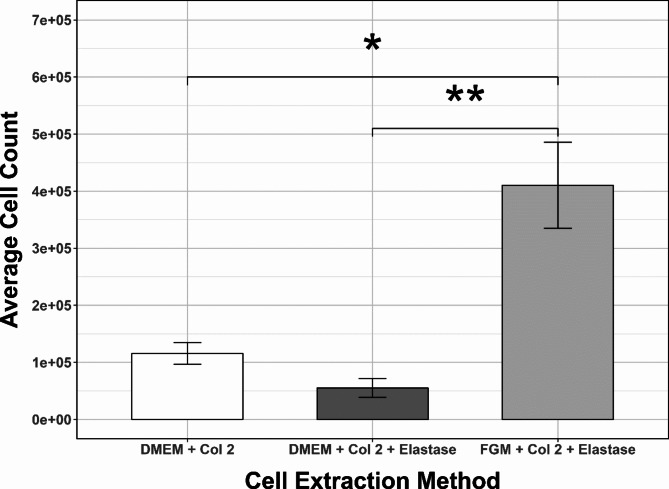
Average cell count of combined cell cultures from R49, R51 and R56 in technical triplicate under three different cell extraction conditions. Results of Mann-Whitney pairwise u-tests for the significance of digestion technique on wing punch cell number acquisition. The P-value was assessed at the two tailed 0.05 significance level after FDR correction. A statistically significant difference *p* = 0.0015 is found between DMEM + Collagenase 2 + Elastase and FGM + Collagenase 2 + Elastase. A statistically significant difference of *p* = 0.0324 is found between DMEM + Collagenase 2 and FGM + Collagenase 2 + Elastase. Error bars represent the standard error of the mean of all combined biological and technical repeats within each method.

The relatively low cell numbers obtained from the bat wing punch biopsies are the key bottleneck of previously published protocols utilizing this tissue type for establishment of primary fibroblasts^9,17^. The performed optimization experiments compared three variants of tissue digestion protocol; including specialised FGM, and addition of elastase to promote release of the cells. High variability in obtained cell yields was observed, both between individuals and between different samples from the same individual (Fig. 1, Supplemental Table 2), however digestion in collagenase and elastase with subsequent culture in fibroblast growth medium resulted in faster confluence and higher cell numbers than digestion in elastase and collagenase followed by culture in DMEM medium the outline of this workflow can be found in Supplementary Fig. 2. This underscores the potential for achieving higher cell yields from the bat wing punch biopsies by introducing modifications to protocol used for tissue digestion and establishment of primary fibroblast culture.

### Immortalisation of primary cultures

**Fig. 2:**
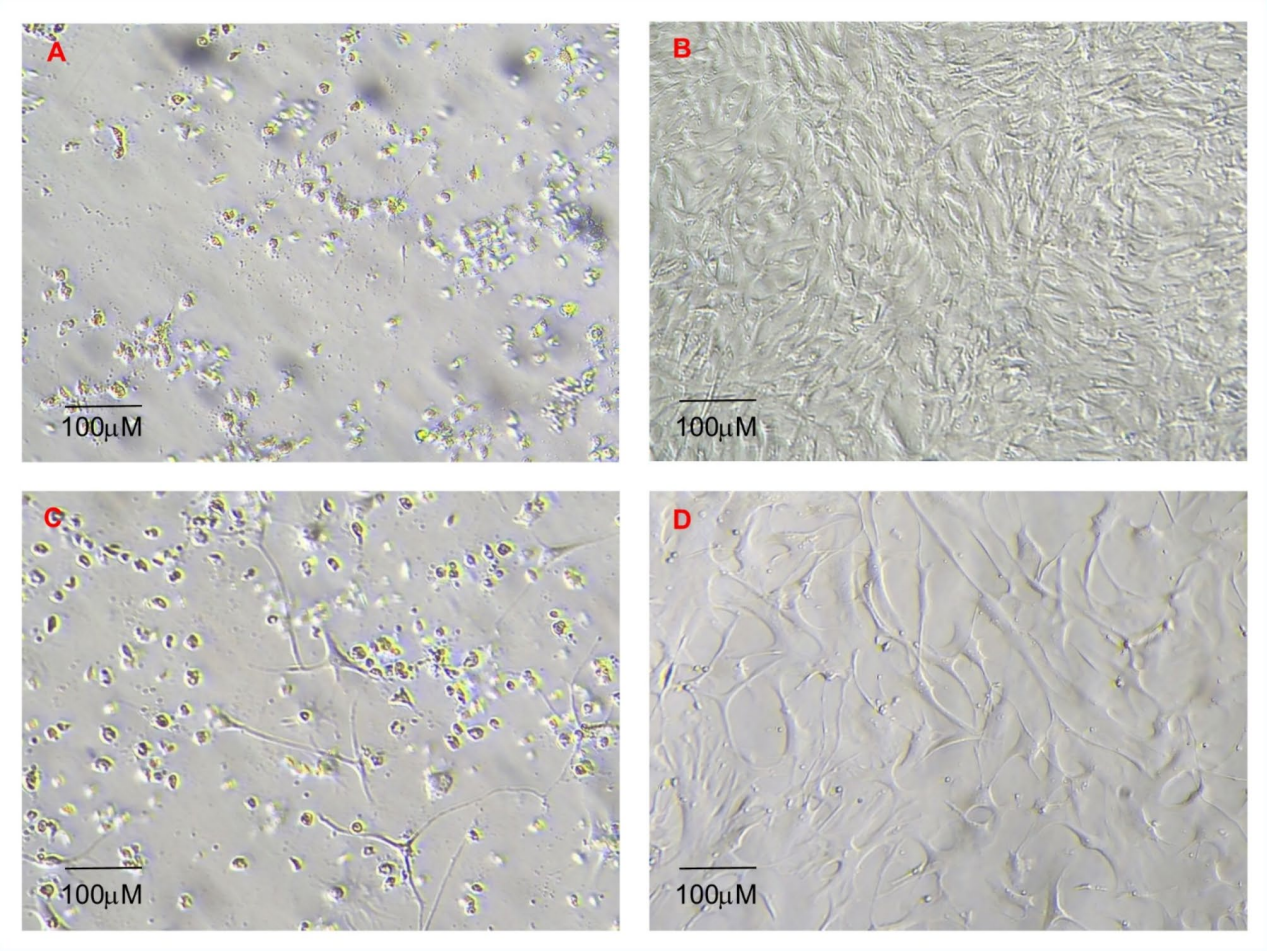
Microscope images of *R.aegyptiacus* cells that have been exposed to different combinations of viruses and antibiotics to generate transformed lines. (**A**) Microscope image of R49 (*R. aegyptiacus*) cell culture that has been exposed to Puromycin only in order to ensure the antibiotic can kill untransformed cells, exposure results in death of all cells as indicated by the absence of adherent fibroblast cells and presence of floating dead cells; (**B**) Microscope image of R49 that has been exposed to the SV40LT procedure as in Image A but with no Puromycin thus showing cell death in A is due to its antibiotic sensitivity for this bat cell line; (**C**) Microscope image of R49 that has been transformed via SV40LT and selected for using Puromycin six days post initial infection. The live fibroblasts that have been transformed and are thus resistant to Puromycin can be clearly seen in contrast to the killed floating dead cells; (**D**) Microscope image of R49 that has been transformed via SV40LT, 14 days post initial infection.

Increased availability of immortalised cell lines from multiple individuals of wide range of bat species would benefit in vitro studies of bat biology. As wing punch biopsies can be collected non-lethally, are minimally invasive to collect, and therefore can be acquired even from protected, wild bat populations, we tested the suitability their suitability for establishment immortalised cell lines. Immortalisation of wing punch derived primary fibroblasts (individuals R42, R46 and R49) was successfully achieved using the LV40LT transformation performed at the MOI (Multiplicity of infection) of 10, 20 and 30 (Fig. 2). The transformation was confirmed as four to five days post antibiotics selection (one week after transduction), all cells in the antibiotic control well were dead (Fig. 2a). Cells in the polybrene control were alive and confluent, the same was also true for the ‘no Puromycin’ control (Fig. 2b). The wells containing cells that had been successfully transduced had a small number of individual cells alive (Fig. 2c). For the transformed cells 10 to 14 days of cell culture time post transfection led to small islands of cells that were then visible (Fig. 2d). It was noted that it was important not to passage to a T25 at less than 70% confluency as this resulted in slow cell growth and viability.

##  Comparisons of primary cells and immortalised cells lines

While immortalised cell lines show robust growth and are available in large numbers making them most suitable for repeatable cellular functional assays, their responses may not completely mimic those of primary cell cultures and therefore caution is required when working with transformed. Therefore, the properties of the primary (R42, R49) and immortalised (R42IM, R49IM) cells derived from the same individuals were examined, in terms of cellular proliferation, maximum passage number achieved, responses to oxidative stress, and activation by TLR agonists.

### Cellular proliferation

**Fig. 3:**
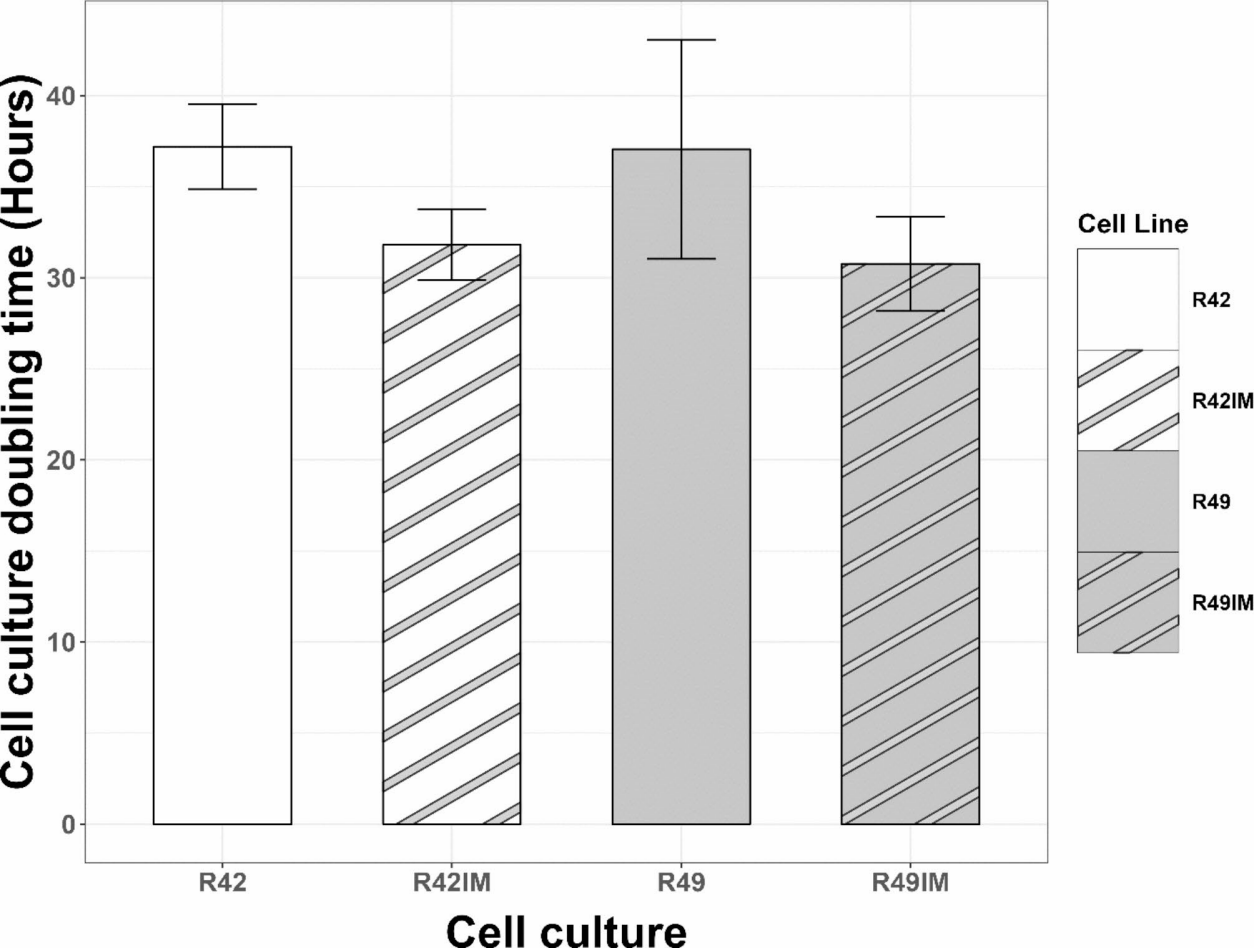
Average doubling time of *R.aegyptiacus* cell lines assessed through the MTT assay. For each comparison of immortalised cell lines (R42IM & R49IM) to their wild type versions there was no statistically significant difference at *p* = 0.05 using a two tailed t-test, R42 p value = 0.30, R49 *p* = 0.28. Error bars represent the standard error of the mean of the biological replicates.

The cell proliferation was assessed via the MTT assay, demonstrating the average doubling time of *R.aegyptiacus* primary fibroblasts of 37 h compared to 31 h in the respective immortalised cell lines at passage 10 (Fig. 3). However, this difference was not statistically significant (*p* = 0.30 for R42 and *p* = 0.28 for R49) suggesting that proliferation of primary and immortalised cells is comparable at the passage examined.

### Maximum passage number

Primary and immortalised cultures were cultured continuously. Untransformed primary fibroblasts (R42 and R49) reached replicative senescence at P18 and 20 respectively, as indicated by the inability to reach confluency within 2 weeks, and visual typical senescence associated morphology of enlarged cell size and flattened appearance. In contrast, immortalised R42IM and R49IM, show no signs of slower proliferation or morphological changes at passages P52 and P60 when the experiment was discontinued.

### Oxidative stress assay

**Fig. 4:**
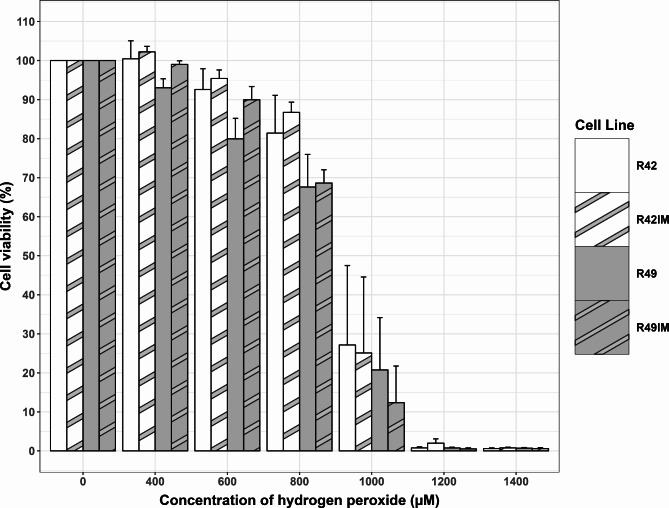
Cell viability of *R.aegyptiacus* cell lines assessed through the MTT assay during the oxidative stress assay which exposed cells to 24 h of varying concentrations of hydrogen peroxide, for each comparison of immortalised cell lines (R42IM & R49IM) to their wild type versions there was no statistically significant difference in the loss of viability at any of the concentration points with a threshold of *p* = 0.05 due to a calculated empirical p value of 1 following testing with 5000 permutations. The effect of oxidative stress exposure indicates an expected decrease in cell viability with increasing concentration with terminal effects being seen at the 1000 µM and above range; the 2000 µM data point has been excluded to enhance clarity. Error bars represent the standard error of the mean of the converted percentage biological replicates. Average LD50s of each cell culture upon H_2_O_2_ treatment were: R42 = 906 µM, R42IM = 912 µM, R49 = 845 µM, R49IM = 855 µM. Following ANOVA at a p threshold of 0.05 with Tukey’s correction, there was no statistically significant difference in the average LD50s between any of the cell cultures during the stress response.

Oxidative stress is a critical factor in cellular ageing and inflammation, pathways that have likely undergone evolutionary adaptations in bats^30^. To ascertain if the immortalisation process does not significantly affect cell sensitivity to oxidative stress, H_2_O_2_ stress treatment was performed. Both primary and immortalised cells showed comparable survival response to H_2_O_2_ (Average LD50 for Primary Lines = 880µM, Immortalised = 883µM) (Fig. 4). There was no significant difference between the cell lines between immortalised and their primary counterparts in response to H_2_O_2_ stress at the different dosages (p value = 1 following testing with 5000 permutations) nor LD50s (as calculated using the drc package within R) between any of the cultures (at a *p* < 0.05 threshold after Tukey corrections using ANOVA). This suggests that immortalisation does not change oxidative stress survival until at least passage 10.

### TLR agonist assay

**Fig. 5:**
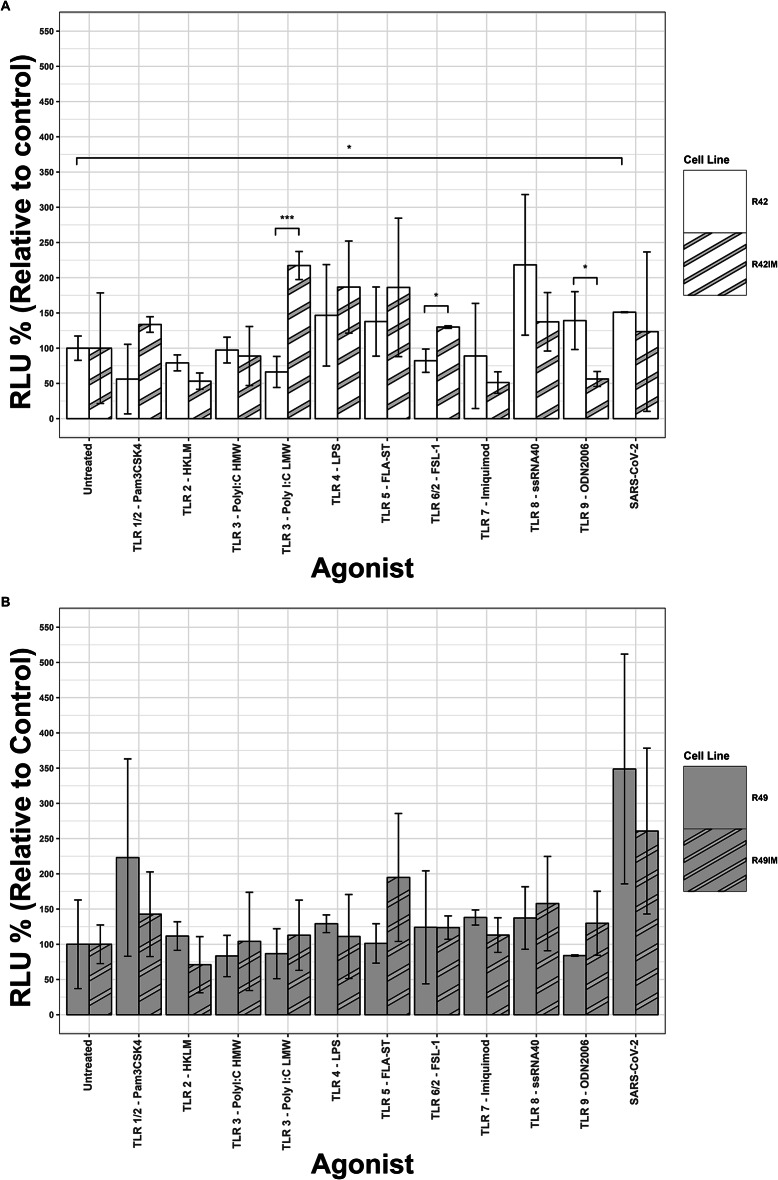
NF-κB response of *R.aegyptiacus* cells to TLR agonists and SARS-CoV-2. Cells from *R.aegyptiacus* individuals R42 (**A**) and R49 (**B**) were transfected with a luciferase tagged TLR reporter and treated with a panel of TLR agonists, or heat inactivated SARS-CoV-2, for 24 h. Primary cells responded similarly to their immortalised counterparts, with statistically significant differences observed between R42 and R42IM in response to TLR3 (Poly(I: C) LMW) and TLR 6/2 (FSL-1); however neither of these responses were significantly increased compared to untreated controls. Heat inactivated SARS-CoV-2 stimulated TLR activity compared with untreated controls in all cells tested, with a statistically significant increase in R42 cells using ANOVA with Tukey’s for multiple comparison correction. Data are representative of three replicates, where ***: *p* < 0.001 and *: *p* < 0.05, with error bars representing ± 1 standard deviation of the mean.

The TLR agonist assay was performed to compare the immune response of fibroblasts and immortalised cell lines. Both primary fibroblasts and their corresponding immortalised cell lines demonstrated modest induction in NF-κB activity in response to the TLR agonist panel, relative to the untreated control (Fig. 5). In general, TLR agonists induced similar responses in primary fibroblasts compared with their immortalised counterparts. In R42 cells compared with R49IM cells, there was a significant difference between the magnitude of their response to TLR 3 (HMW poly(I: C)), TLR 6/2 (FSL-1) and TLR 9 - (ODN2006); however, neither cell type had significantly increased responses to these TLRs relative to the untreated controls. Interestingly, heat-inactivated SARS-CoV-2 induced a significant activation of NF-κB in R42 cells compared to untreated control, while R49 cells and immortalised cell lines had a more modest increase in TLR activity in response to heat-inactivated SARS-CoV-2 that did not reach statistical significance.

## Species identification

In order to ensure that the cell lines derived from *R.aegyptiacus* we PCR amplified Cytochrome b and performed sanger sequencing from each cell line. All sequences generated ’blasted’ ~ 100% to previously published *R.aegyptiacus* cytochrome b sequences, therefore confirming species identity (Supplementary Table 3).

## Discussion

The lack of cellular resources has been a major stumbling block for developing bats as model systems. While primary and immortalised bat cell lines have been created by virologists to explore the viral resistance of bat cells from multiple species^42–44^, these are usually derived from lethally sampled organs (Supplemental Table 1). Here we have optimised protocols for establishing primary fibroblasts and immortalised cell lines from 3 mm bat wing membrane biopsies, a sample type that can be non-lethally acquired from wild and protected bats. Therefore, our methods have the potential to expand the taxonomic representation of bat species in cellular assays.

Our improved protocol to isolate and culture primary fibroblast cells from bat wing punches from *R.aegyptiacus* demonstrates that a modified digestion procedure (inclusion of elastase enzyme) in combination with fibroblast specific growth medium (FGM) can increase the yield of primary cells obtained from individual wing biopsy. Interestingly, elastase had no significant effect on yields of cells growing in standard DMEM medium. Further experimentation is therefore required to ascertain how different concentrations of elastase impact the number of cells released during bat-wing tissue digestion, subsequent cell attachment and growth, to establish the optimal proliferation protocol across different bat species, and achieve maximum fibroblasts yield per wing punch.

Typically, the bat wing membrane is composed of two thin layers of dermis containing nerves, muscle fibres, blood vessels and a network of connective tissue fibre bundles. Indeed, this architecture has been shown to differ between species. Bat species can vary massively in size, ecological niche, and sensory perception, and wing form and function may be related to diet, size, and migration habits, among other factors^45^. Thickness of the wing has shown a weak positive correlation with body size^46^, and this alone is likely to cause a variation in cell numbers recovered from biopsies using the methods described in this study. Additionally, there are limited studies on the histology of bat wing membranes and how this may differ between bat taxa^35,47^. Further investigation into the culture methods described here is needed to evaluate its application to other species using non-lethally acquired wing biopsies. These results underscore the potential to maximize the cell yield obtained with adjustments of the digestion protocol and should be considered when generating primary bat cell cultures from wing biopsies.

Further, fibroblasts generated from wing punch biopsies, were successfully immortalised using a modified SV40LT. The generated cell lines continued to proliferate until passage 46 and more without noticeable morphological changes, whereas primary fibroblasts showed signs of senescence such as flat and enlarged shape, rough cell edges and much longer doubling times at passages 18–20. Cellular immortalisation, while capable of generating an extensive supply of cells, can result in cells with different cellular properties to primary cells e.g. an increased tolerance to oxidative damage^48^, enhanced proteostasis, endoplasmic reticulum function and calcium signalling^49^. Consequently, caution is often required when interpreting results obtained using immortalised cell lines rather than primary cells. However, in this study we showed no significant differences in cell proliferation nor survival response to hydrogen peroxide induced oxidative stress between the primary and immortal cell lines derived from the same individuals, suggesting that immortalised bat cell lines responded like primary cells at passages studied. The similar proliferation rate between wild type and immortal lines has been reported in bats before^50^, but whether this observation needs to be further investigated as only two immortalised cell lines were examined in this study. The effect of the age of the individual sampled on cell proliferation also needs to be investigated, as all fibroblast cell lines here were generated from juveniles, and potentially there could be a differential proliferation and response rate between older and younger cells^9,51^. Nevertheless, our data show that immortalised cell lines derived from wing punch biopsies offer a promising tool for studying oxidative stress and other cellular responses in bats though further experiments to show the mechanisms would be required such as with transcriptomics, antioxidant assays or DNA damage marker 8-OHdG.

Additionally, we compared the response of primary and immortalised cells to TLR agonists. Due to bats’ exceptional immunity and their suspected tolerance of disease from infections^52^, developing bat cell lines suitable for exploring the mechanisms underlying their unique immunity is required^11^ and it is important to characterise any differences arising from the process of immortalisation itself. This is arguably even more so for the purposes of immunological investigation as there is a well-established close link between antiviral defence and tumour suppression^53^. Here we used a NF-κB luciferase reporter assay to test the NF-κB response to a variety of TLR agonists, as well as inactivated SARS-CoV-2. However, both primary and immortalised cells derived from two individuals showed only a modest response to used TLR agonists, that was generally not statistically significant. High variability of the observed response indicates the need for testing cells derived from larger number of individuals, and potentially including other immune assays, but the fact that primary and immortalised cells derived from the same individual demonstrate similar response in terms of magnitude of relative NF-κB induction, suggests that the immortalisation protocol itself did not significantly change the profile of immune response of the bat wing fibroblasts.

In conclusion, we have developed an optimised protocol for the establishment of primary bat fibroblast cultures obtained through minimally invasive wing punches from *R.aegyptiacus*. Further, we successfully generated multiple immortalised cell lines using the SV40LT on wing punch biopsy derived fibroblast cells. This represents the first instance of an immortal cell line being generated in bats using a repeatable non-lethal sampling method. The immortalised cell lines retained the properties as their original primary cells in terms of cell proliferation rates at passage 10, oxidative stress tolerance and response to the TLR agonists. This study represents the first step towards expanding resources for scientists interested in studying the cellular basis of unique evolutionary adaptations in bats. Given these protocols we can now non-lethally sample bats at the population level and develop both primary and immortal cellular resources from multiple individuals and multiple species, without sacrificing a single individual. This will enable the cellular experiments required to uncover the molecular regulators of bats’ extended healthspan and other unique traits, and to develop bats as new model study species and systems.

## Electronic supplementary material

Below is the link to the electronic supplementary material.

Supplementary Material 1

## Data Availability

All data generated or analysed during this study are included in this published article and supplementary files.

## References

[1] Simmons, N. B. & Cirranello, A. L. Bat Species of the World: A taxonomic and geographic database. *Bat species of the world: A taxonomic and geographic database.* Version 1.6. Accessed on 29/09/2024. (2024). https://batnames.org/

[2] Teeling, E. C. et al. Bat Biology, genomes, and the Bat1K project: to generate chromosome-level genomes for all living bat species. *Annu. Rev. Anim. Biosci.* **6**, 23–46 (2018). 29166127 10.1146/annurev-animal-022516-022811

[3] Wilkinson, G. S. & Adams, D. M. Recurrent evolution of extreme longevity in bats. *Biol. Lett.* **15**, 20180860 (2019). 30966896 10.1098/rsbl.2018.0860 PMC6501359

[4] Wilkinson, G. S. & South, J. M. Life history, ecology and longevity in bats. *Aging Cell.* **1**, 124–131 (2002). 12882342 10.1046/j.1474-9728.2002.00020.x

[5] Banerjee, A. et al. Novel insights into Immune systems of bats. *Front. Immunol.* **11**, 26 (2020). 32117225 10.3389/fimmu.2020.00026 PMC7025585

[6] Irving, A. T., Ahn, M., Goh, G., Anderson, D. E. & Wang L.-F. lessons from the host defences of bats, a unique viral reservoir. *Nature*. **589**, 363–370 (2021). 33473223 10.1038/s41586-020-03128-0

[7] Foley, N. M. et al. Growing old, yet staying young: the role of telomeres in bats’ exceptional longevity. *Sci. Adv.* **4**, eaao0926 (2018). 29441358 10.1126/sciadv.aao0926 PMC5810611

[8] Huang, Z. et al. Longitudinal comparative transcriptomics reveals unique mechanisms underlying extended healthspan in bats. *Nat. Ecol. Evol.* **3**, 1110–1120 (2019). 31182815 10.1038/s41559-019-0913-3

[9] Kacprzyk, J. et al. Evolution of mammalian longevity: age-related increase in autophagy in bats compared to other mammals. *Aging*. **13**, 7998–8025 (2021). 33744862 10.18632/aging.202852 PMC8034928

[10] Moreno Santillán, D. D. et al. Large-scale genome sampling reveals unique immunity and metabolic adaptations in bats. *Mol. Ecol.* **30**, 6449–6467 (2021). 34146369 10.1111/mec.16027

[11] Wang, L. F., Gamage, A. M., Chan, W. O. Y., Hiller, M. & Teeling, E. C. Decoding bat immunity: the need for a coordinated research approach. *Nat. Rev. Immunol.* **21**, 269–271 (2021). 33649605 10.1038/s41577-021-00523-0 PMC7919622

[12] Gonzalez, V. & Banerjee, A. Molecular, ecological, and behavioral drivers of the bat-virus relationship. *iScience*. **25**, 104779 (2022). 35875684 10.1016/j.isci.2022.104779 PMC9296223

[13] Ahn, M., Cui, J., Irving, A. T. & Wang, L. F. Unique loss of the PYHIN gene family in bats amongst mammals: implications for inflammasome sensing. *Sci. Rep.* **6**, 21722 (2016). 26906452 10.1038/srep21722 PMC4764838

[14] Jebb, D. et al. Six reference-quality genomes reveal evolution of bat adaptations. *Nature*. **583**, 578–584 (2020). 32699395 10.1038/s41586-020-2486-3 PMC8075899

[15] Huang, Z., Whelan, C. V., Dechmann, D. & Teeling, E. C. Genetic variation between long-lived versus short-lived bats illuminates the molecular signatures of longevity. *Aging*. **12**, 15962–15977 (2020). 32674072 10.18632/aging.103725 PMC7485743

[16] Shen, Y. Y. et al. Adaptive evolution of energy metabolism genes and the origin of flight in bats. *Proc. Natl. Acad. Sci.* 107, 8666–8671 (2010). 10.1073/pnas.0912613107 PMC2889356 20421465

[17] Kacprzyk, J., Teeling, E. C., Kelleher, C. & Volleth, M. Wing membrane biopsies for bat cytogenetics: finding of 2n = 54 in Irish rhinolophushipposideros (*Rhinolophidae, Chiroptera, Mammalia*) supports two geographically separated chromosomal variants in Europe. *Cytogenet. Genome Res.* **148**, 279–283 (2016). 27333200 10.1159/000447111

[18] Yohe, L. R. et al. Tissue collection of bats for omics analyses and primary cell culture. *J. Vis. Exp.* **59505**10.3791/59505 (2019). 10.3791/59505 31710024

[19] Shay, J. W. & Wright, W. E. Hayflick, his limit, and cellular ageing. *Nat. Rev. Mol. Cell. Biol.* **1**, 72–76 (2000). 11413492 10.1038/35036093

[20] Stewart, S. A. & Weinberg, R. A. Senescence: does it all happen at the ends? *Oncogene*. **21**, 627–630 (2002). 11850788 10.1038/sj.onc.1205062

[21] Kunieda, T. et al. Transduction of immortalized human hepatocytes with p21 to enhance differentiated phenotypes. *Cell. Transpl.* **11**, 421–428 (2002). 12382668

[22] Mayne, L. V., Priestley, A., James, M. R. & Burke, J. F. Efficient immortalization and morphological transformation of human fibroblasts by transfection with SV40 DNA linked to a dominant marker. *Exp. Cell. Res.* **162**, 530–538 (1986). 3002824 10.1016/0014-4827(86)90356-3

[23] Steele, S. L. et al. Telomerase immortalization of principal cells from mouse collecting duct. *Am. J. Physiol. Ren. Physiol.* **299**, F1507–F1514 (2010). 10.1152/ajprenal.00183.2010 PMC3006301 20926633

[24] Koh, J. et al. ABCB1 protects bat cells from DNA damage induced by genotoxic compounds. *Nat. Commun.* **10**, 2820 (2019). 31249297 10.1038/s41467-019-10495-4 PMC6597548

[25] Maeda, K. et al. Isolation of novel adenovirus from fruit bat (*Pteropus dasymallus yayeyamae*). *Emerg. Infect. Dis. J.* **14**, 347 (2008). 10.3201/eid1402.070932 PMC2630045 18258142

[26] Kobayashi, T. et al. Characterization of a novel species of adenovirus from Japanese microbat and role of CXADR as its entry factor. *Sci. Rep.* **9**, 573 (2019). 30679679 10.1038/s41598-018-37224-z PMC6345744

[27] Ahn, M. et al. Dampened NLRP3-mediated inflammation in bats and implications for a special viral reservoir host. *Nat. Microbiol.* **4**, 789–799 (2019). 30804542 10.1038/s41564-019-0371-3 PMC7096966

[28] Elbadawy, M. et al. Establishment of intestinal organoid from *Rousettus Leschenaultii* and the susceptibility to bat-associated viruses, SARS-CoV-2 and *Pteropine Orthoreovirus*. *Int. J. Mol. Sci.* **22**, 10763 (2021). 34639103 10.3390/ijms221910763 PMC8509532

[29] Pride, H. et al. Long-lived species have improved proteostasis compared to phylogenetically-related shorter-lived species. *Biochem. Biophys. Res. Commun.* **457**, 669–675 (2015). 25615820 10.1016/j.bbrc.2015.01.046

[30] Harper, J. M., Salmon, A. B., Leiser, S. F., Galecki, A. T. & Miller, R. A. Skin-derived fibroblasts from long-lived species are resistant to some, but not all, lethal stresses and to the mitochondrial inhibitor rotenone. *Aging Cell.* **6**, 1–13 (2007). 17156084 10.1111/j.1474-9726.2006.00255.x PMC2766812

[31] Bertelsen, M. F. 23—Issues surrounding surplus animals in zoos. In Fowler’s Zoo and Wild Animal Medicine Current Therapy, Volume 9 (eds Miller, R. E., Lamberski, N. & Calle, P. P.) 134–136 (W.B. Saunders, doi:10.1016/B978-0-323-55228-8.00023-0. (2019).

[32] Weaver, K. N., Sara, E., Alfano, Amanda, R., Kronquist & DeeAnn, M. Reeder. Healing rates of wing punch wounds in free-ranging little brown Myotis (*Myotis lucifugus*). *Acta Chiropterologica*. **11**, 220–223 (2009).

[33] Greville, L. J., Ceballos-Vasquez, A., Valdizón-Rodríguez, R., Caldwell, J. R. & Faure, P. A. Wound healing in wing membranes of the Egyptian fruit bat (*Rousettus aegyptiacus*) and big brown bat (*Eptesicus fuscus*). *J. Mammal*. **99**, 974–982 (2018).

[34] Power, S. *Non-Lethal Cell Culture in the Order Chiroptera: Implications for Viral Studies* (University College Dublin. School of Biology and Environmental Science, 2021).

[35] Holbrook, K. A. & Odland, G. F. A collagen and elastic network in the wing of the bat. *J. Anat.* **126**, 21–36 (1978). 649500 PMC1235709

[36] Crowley, G. & Hall, L. Histological observations on the wing of the grey-headed flying-fox (*Pteropus-Poliocephalus*) (*Chiroptera, Pteropodidae*). *Aust J. Zool.* **42**, 215–231 (1994).

[37] R Core Team. *R: A Language and Environment for Statistical Computing* (R Foundation for Statistical Computing, 2022).

[38] Atkuru, S. et al. Cellular ageing of oral fibroblasts differentially modulates extracellular matrix organization. *J. Periodontal Res.* **56**, 108–120 (2021). 32969036 10.1111/jre.12799

[39] Ritz, C., Baty, F., Streibig, J. C. & Gerhard, D. Dose-response analysis using R. *Plos One*. **10**, e0146021 (2015). 26717316 10.1371/journal.pone.0146021 PMC4696819

[40] Fletcher, N. F. et al. Activated macrophages promote hepatitis C virus entry in a tumor necrosis factor-dependent manner. *Hepatol. Baltim. Md* 59, 1320–1330 *PLOS ONE* 10, e0146021 (2015). (2014). 10.1002/hep.26911 PMC4255687 24259385

[41] Puechmaille, S. J. et al. The evolution of sensory divergence in the context of limited gene flow in the bumblebee bat. *Nat. Commun.* **2**, 573 (2011). 22146392 10.1038/ncomms1582 PMC3247819

[42] Crameri, G. et al. Establishment, immortalisation and characterisation of pteropid bat cell lines. *PLoS ONE*. **4**, e8266 (2009). 20011515 10.1371/journal.pone.0008266 PMC2788226

[43] Brook, C. E. et al. Accelerated viral dynamics in bat cell lines, with implications for zoonotic emergence. *eLife*. **9**, e48401 (2020). 32011232 10.7554/eLife.48401 PMC7064339

[44] Aicher, S. M. et al. Species-specific molecular barriers to SARS-CoV-2 replication in bat cells. *J. Virol.* **96**, e00608–e00622 (2022). 35862713 10.1128/jvi.00608-22 PMC9327701

[45] Swartz, S. M., Groves, M. S., Kim, H. D. & Walsh, W. R. Mechanical properties of bat wing membrane skin. *J. Zool.* **239**, 357–378 (1996).

[46] Studier, E. H. Some physical properties of the wing membranes of bats. *J. Mammal*. **53**, 623–625 (1972).

[47] Cheney, J. A., Konow, N., Bearnot, A. & Swartz, S. M. A wrinkle in flight: the role of elastin fibres in the mechanical behaviour of bat wing membranes. *J. R Soc. Interface*. **12**, 20141286 (2015). 25833238 10.1098/rsif.2014.1286 PMC4424667

[48] Kondoh, H., Lleonart, M. E., Bernard, D. & Gil, J. Protection from oxidative stress by enhanced glycolysis: a possible mechanism of cellular immortalization. *Histol. Histopathol*. **22**, 85–90 (2007). 17128414 10.14670/HH-22.85

[49] Mazzucchelli, G. D. et al. Proteome alteration induced by hTERT transfection of human fibroblast cells. *Proteome Sci.* **6**, 12 (2008). 18419814 10.1186/1477-5956-6-12 PMC2386453

[50] Banerjee, A. et al. Generation and characterization of *Eptesicus fuscus* (big brown bat) kidney cell lines immortalized using the *Myotis Polyomavirus* large T-antigen. *J. Virol. Methods*. **237**, 166–173 (2016). 27639955 10.1016/j.jviromet.2016.09.008 PMC7113758

[51] Cristofalo, V. J., Beck, J., Allen, R. G. & Commentary Cell senescence: an evaluation of replicative senescence in culture as a model for cell aging in situ. *J. Gerontol. Ser. A*. **58**, B776–B779 (2003). 10.1093/gerona/58.9.b776 14528030

[52] Mandl, J. N., Schneider, C., Schneider, D. S. & Baker, M. L. Going to bat(s) for studies of disease tolerance. *Front. Immunol.* **9**, (2018). 10.3389/fimmu.2018.02112 PMC6158362 30294323

[53] Hare, D., Collins, S., Cuddington, B. & Mossman, K. The importance of physiologically relevant cell lines for studying virus-host interactions. *Viruses*. **8**, 297 (2016). 27809273 10.3390/v8110297 PMC5127011

