# Publisher Correction: Generating bat primary and immortalised cell-lines from wing biopsies

**DOI:** 10.1038/s41598-024-82834-5

**Published:** 2024-12-17

**Authors:** Dominic Alcock, Sarahjane Power, Bridget Hogg, Carlotta Sacchi, Joanna Kacprzyk, Sarah McLoughlin, Mads Frost Bertelsen, Nicola F. Fletcher, Aidan O’Riain, Emma C. Teeling

**Affiliations:** 1 UCD School of Biology and Environmental Science, University College Dublin, Dublin, Ireland; 2 UCD School of Veterinary Medicine, Veterinary Science Centre Belfield, University College Dublin, Dublin, Ireland; 3 Center for Zoo and Wild Animal Health, Copenhagen Zoo, Frederiksberg, Denmark; 4 UCD School of Veterinary Medicine, UCD Conway Institute, University College Dublin, Dublin, Ireland

Correction to: *Scientific Reports* 10.1038/s41598-024-76790-3, published online 12 November 2024

In the original version of this Article, Dominic Alcock, Sarahjane Power and Bridget Hogg were omitted as equally contributing authors.

The original Article has been corrected.

