# Supplementary material for: Generating bat primary and immortalised cell-lines from wing biopsies
Source: Sci Rep. 2024 Nov 12;14:27633. doi: 10.1038/s41598-024-76790-3 (PMC11555217; doi:10.1038/s41598-024-76790-3)
Supplement: Supplementary file 1 — Supplementary Material 1 [file 41598_2024_76790_MOESM1_ESM.docx]

Supplementary Information

**Supplementary Text S1:**

**Transduction Protocol:**

1. Prepare DMEM complete with 10 mg/ml polybrene (Sigma-Aldrich: TR-1003-G)
2. Prepare 7 ml of media containing 50,000 cells per ml or 350,000 cells in total in DMEM complete plus polybrene per six well plate. The final seeding density will be 50,000 cells per well of a six well plate.
3. Remove the Lentivirus from the –80^o^C freezer and rapidly thaw at 37^o^C. If all the virus is not being used aliquot into smaller batches for downstream use as repeated freeze thaw reduces virus efficiency.
4. Prepare 500 μl of virus at a Multiplicity of Infection (MOI) of 10, 20 and 30 for each well to be treated using the following formula:

Virus Volume (ml) = MOI x cell number

Virus Titer (IU/ml)

For example: if the seeding density was 50,000 the Virus Titre was 1x10^9^/ml and an MOI of 30 was required then:

Virus Volume (ml) = 30 x 50,000 = 0.0015 ml

1,000,000,000

1. Add 500 μl of a single viral dilution to one well of a six well plate.
2. Into two wells add 500 μl of DMEM complete plus polybrene. These wells will be used to verify antibiotic activity (antibiotic control) and that the polybrene (polybrene control) is not having any adverse effect on the cells.
3. Perform a “reverse transduction” by adding 1 ml of cells, 50,000 cells/ml, to each well containing the viral dilution. Also add 1 ml of cells, 50,000 cells/ml, just containing DMEM complete plus polybrene.
4. Incubate at 37^o^C 5 % CO_2_.
5. After 48 h replace the media in the wells containing the virus and one of the wells containing just DMEM polybrene (antibiotic control) with DMEM complete 0.5 μg/ml Puromycin. Replace the media in the other well with just DMEM polybrene with DMEM and no antibiotic (polybrene control).
6. After 48 h replace the media in the wells containing DMEM complete 0.5 μg/ml Puromycin with DMEM complete 1 μg/ml Puromycin. In the well containing the polybrene control, replace the media with DMEM completely.
7. Change the media every two to three days with fresh DMEM complete with 1 μg/ml Puromycin, or just DMEM complete for the polybrene control, until cell islands have formed in the transduced wells.
8. Once the cells in the transduced wells have reached 70-80% confluency, using 1 ml/well 0.05% Trypsin-EDTA as described above, transfer to a T25 in 4 ml DMEM complete plus 1 μg/ml Puromycin. If islands are forming but not expanding only change 1 ml of the media. Alternatively conditioned media taken from the parental culture grown to 80% confluency can be used in a ratio of 1:4 conditioned to fresh media respectively to aid cell growth.

Transient transformation using ViaFect^TM^:

Visualisation of staining showed fluorescence for both DAPI and Alexa Fluor®488 in the SV40 transduced lines but only fluorescence for DAPI in the parental culture (data not shown) confirming immortalisation with SV40. Both the parental lines and the immortalised lines could be transiently transformed with GFP as demonstrated in Supplemental Figure 1. The optimisation of the transformation showed that the best ratio to use was 500 ng of plasmid DNA to 3 μl of Viafect^TM^. This was therefore the ratio used for transformation with the TLR agonist reporter plasmids.

**Supplemental Figures**


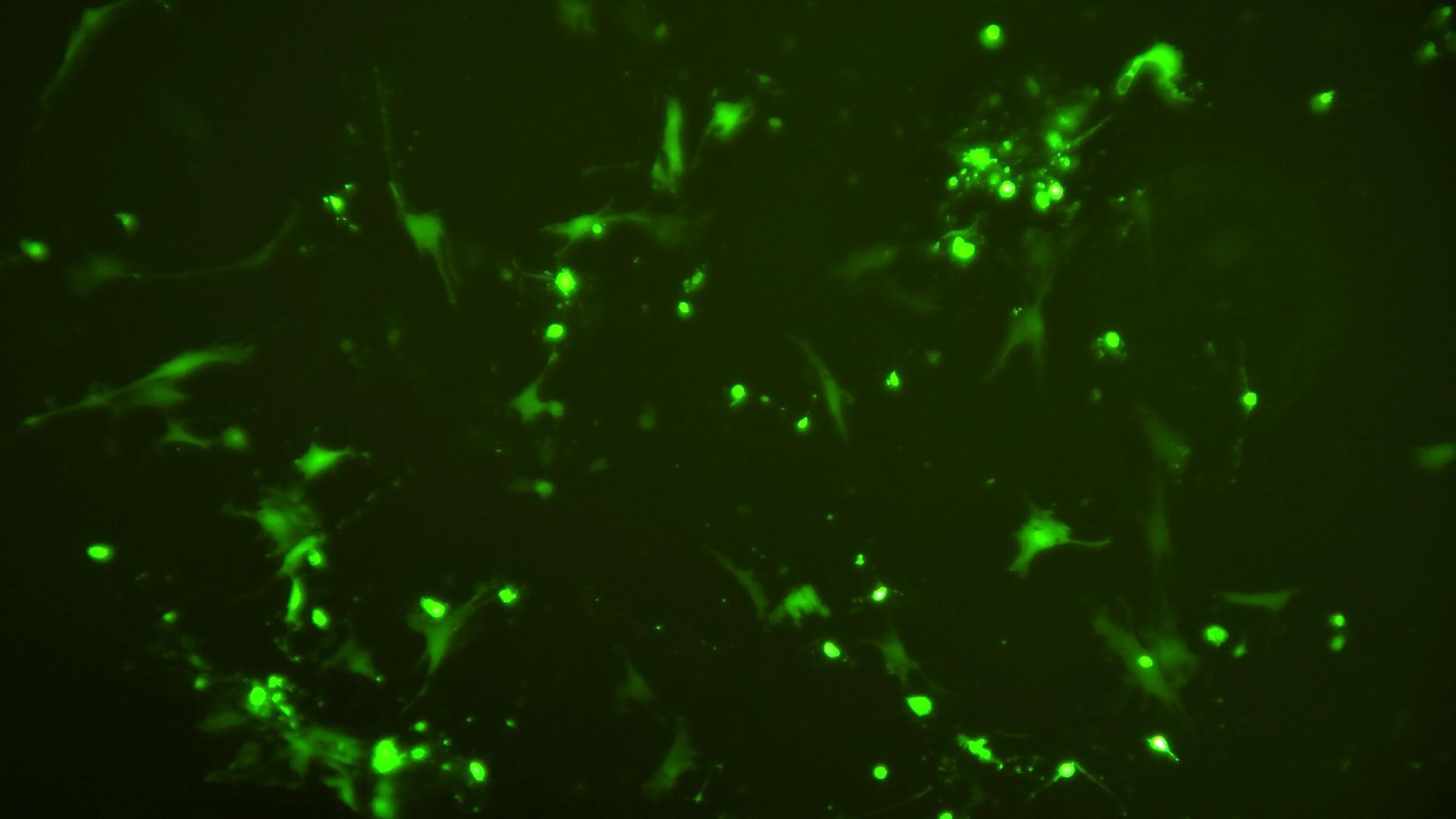

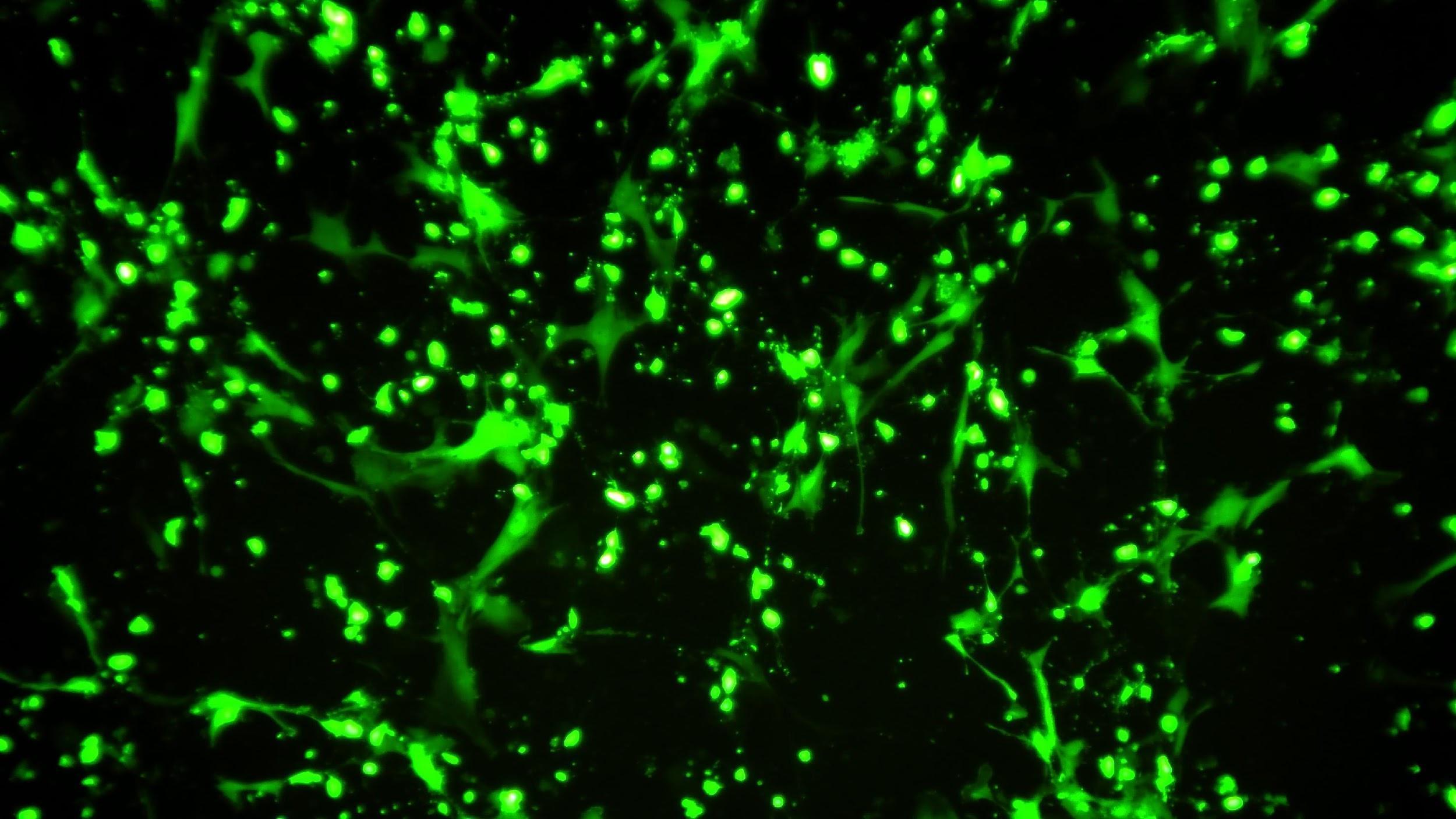


**Supplemental Figure 1:** Panels showing fluorescence microscopy of the R49 cell line subjected to transient transformation with enhanced GFP on the pmaxGFP^TM^ vector with absorbance at 495 nm, R49 wild type cell culture on the left and R49 immortalised cell line on the right.


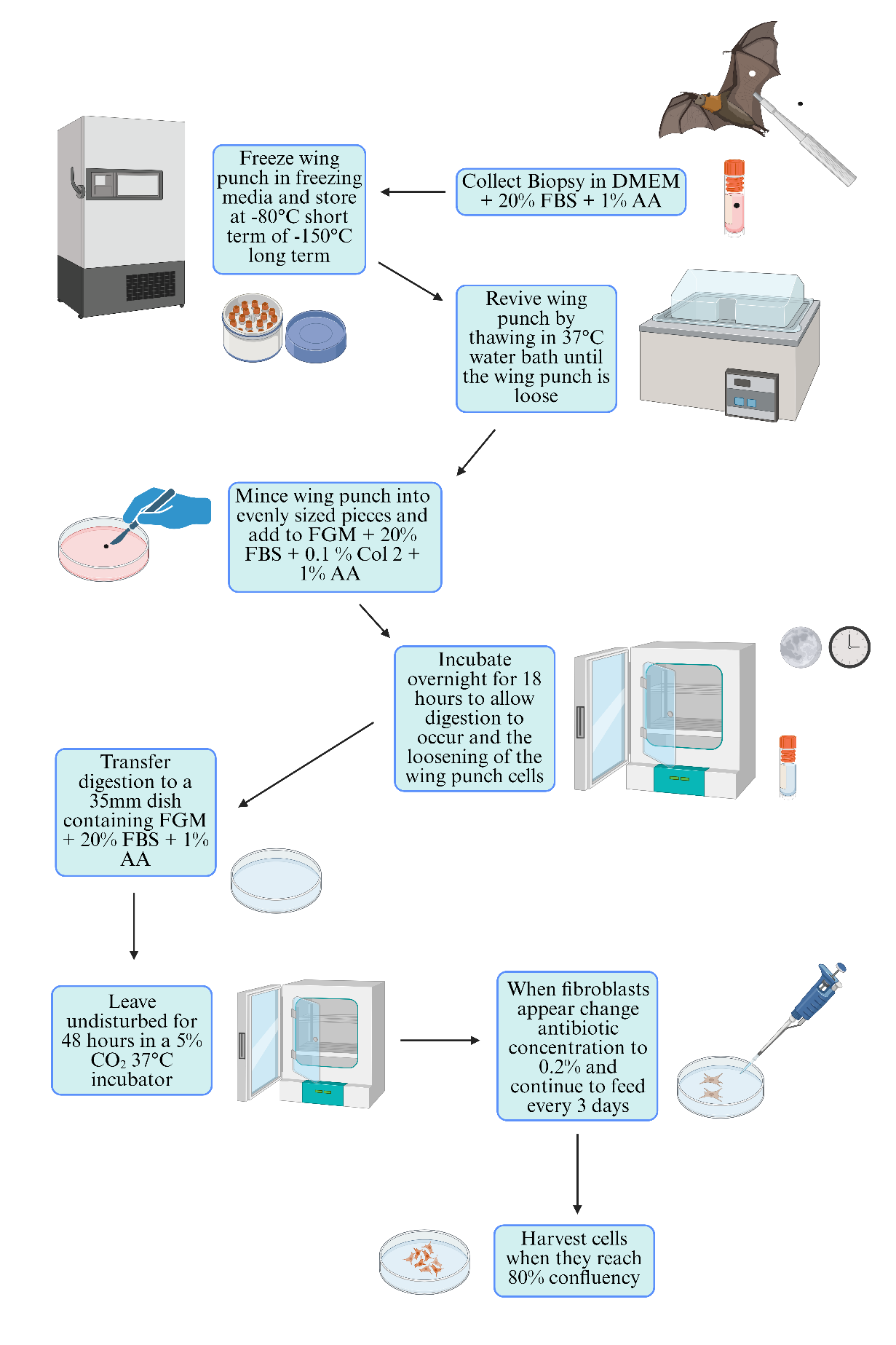


**Supplemental Figure 2:** Image showing workflow of wing punch digestion with optimum technique. Image created in BioRender.com

**Supplemental Tables**

**Supplemental Table 1:** List of published bat cell culture studies to (25/01/2024).

| **Species** | **Tissue Origin** | **Cell Type** | **Primary** | **Immortal** | **Digestion and Initial Culture** | **Medium** | **Reference** |
| --- | --- | --- | --- | --- | --- | --- | --- |
| *Artibeus jamaicensis* | Ovaries | Oocytes / Cortical Germ Cells / Granulosa | ✓ | - | Ovaries: L-15 medium initially followed by digestion in 0.25% trypsin, 0.1 mM ethylene diamine tetra acetic acid (EDTA), 1.6 mg/ml of hyaluronidase and 20 μg/ml DNase in Rinaldini solution at 37°C for 20 min under humid conditions, with 5% carbon dioxide followed by inactivation of enzymes by FBS.  Adult Cortical Germ Cells: The cortical region of the ovary was collected and incubated in 0.25% trypsin in Rinaldini solution and 1 mM of EDTA (Sigma-Aldrich) for 10 min at 37°C. Enzyme activity was subsequently eliminated with 10% FBS in McCoy culture medium and 20 μg/ml DNase. Dissociation of cells was then performed by repeated mixing using a Pasteur pipette. | McCoy’s 5a modified culture medium, containing 10% normal mouse serum, 200 IU/ml penicillin G and 200 μg/ml of streptomycin. | S1 |
| *Artibeus planirostris* | Bone Marrow | - | ✓ | - | Flushed with DMEM and 10% FBS using a needle with contents centrifuged at 1000 rpm for 5 min, resuspended in media and plated in cell culture plates. | DMEM + 10% FBS | S2 |
|  | Ear | Fibroblast | ✓ | - | Tissue was cut into small pieces and then treated with collagenase type I (0,1%) and transferred to an incubator at 37°C for 3 h, cells were then centrifuged and transferred to culture bottles. | - |  |
| *Artibeus sp.* | Ear skin | Fibroblast | ✓ | - | 2 mm biopsy minced into small pieces and transferred to a 25 cm^2^ cell culture bottle, containing culture medium and 50% bovine foetal serum, with a final volume of 1 ml. Culture was incubated at 37°C and the medium changed whenever the nutrients became depleted. Growth was observed with an inverted optical microscope. Once fibroblastic cell growth appeared, the cells were trypsinised and then suspended in a complete culture medium with 10% foetal serum, using the same routine as for cell culture. | MEM GLASGOW, 100 U/ml penicillin, 10-4 g/ml streptomycin, 10-4 g/ml fungizone, and 0.003 g/ml L glutamine | S3 |
| *Carollia perspicillata* | Kidney | - | ✓ | SV40 | - | - | S4 |
|  | Trachea | Epithelial | ✓ | SV40T | Trachea and large bronchi tissues minced and placed in a 6-well cell culture plate and submerged in 37°C warm medium. Cell culture plates were left undisturbed for the first 3 days. Following this, cells were checked daily and when an outgrowth of primary cells was observed, the medium was changed every 2 days. | Primary airway epithelial cell medium basal mix supplemented with bovine pituitary extract 0.004 ml/ml, epidermal growth factor (recombinant human) 10 ng/ml, insulin (recombinant human) 5 µg/ml, hydrocortisone 0.5 µg/ml, epinephrine 0.5 µg/ml, triiodo-L-thyronine 6.7 ng/ml, holo-transferrin (human) 10 µg/ml, and retinoic acid 0.1 ng/ml. Supplemented with penicillin/streptomycin, ofloxacin, and amphotericin B. | S5 |
|  |  | Organoid | ✓ | - | A small piece of bat trachea was washed three times in PBS. The tissue was then chopped and digested with 5 mg/mL protease in DMEM/F12 supplemented with antibiotics overnight. On day two, 5% fetal bovine serum was added to the digestion solution and the sample was passed through a 100 μm cell strainer. Centrifugation at 300 *g* was then performed and single trachea and lung cells were collected and embedded in 60% basement membrane Matrigel. | Organoid growing medium with a 1:1 mixture of L-WNR condition medium and additives EGF (50 ng/mL), A83-01 (500 nM), SB 202190 (10 μM) and B27 supplement. | S6 |
| *Cynopteris brachyotis* | Lung | Fibroblast | ✓ | - | Tissues were sliced into 1 mm sized pieces and incubated in a 0.22 µm filtered collagenase solution at 37°C with rotation (60 rpm) for 1 h to dissociate cells. Dissociated tissues were centrifuged at 430 g for 5 min, resuspended in medium and plated in T-25 culture flasks. Cells were maintained at 37°C with 5% CO_2_ until confluence. After the second passage, all cell lines were grown in medium. | Initial culture - RPMI medium 1640 (10% foetal bovine serum, 2% penicillin/streptomycin, 2.5%  After second passage - DMEM with 10% foetal bovine serum and penicillin/ streptomycin | S7 |
| *Eidolon helvum* | Kidney | - | ✓ | SV40 | Lysis of tissues by mincing followed by digestion using 0.05% Trypsin. | DMEM, 4.5 g/L Glucose, 10% Foetal Bovine Serum, 1% Penicillin/Streptomycin, 1% L-Glutamine 200 mM, 1% Sodium Pyruvate 100 mM, 1% MEM nonessential amino acids. | S8 |
|  | Spleen | - | ✓ | SV40 | - | RPMI-1640 medium with 10% FCS, L-glutamine, and penicillin–streptomycin. | S9 |
|  | Trachea | Epithelial | ✓ | SV40T | Trachea and large bronchi tissues minced and placed in a 6-well cell culture plate and submerged in 37°C warm medium. Cell culture plates were left undisturbed for the first 3 days. Following this, cells were checked daily and when an outgrowth of primary cells was observed, the medium was changed every 2 days. | Primary airway epithelial cell medium basal mix supplemented with bovine pituitary extract 0.004 ml/ml, epidermal growth factor (recombinant human) 10 ng/ml, insulin (recombinant human) 5 µg/ml, hydrocortisone 0.5 µg/ml, epinephrine 0.5 µg/ml, triiodo-L-thyronine 6.7 ng/ml, holo-transferrin (human) 10 µg/ml, and retinoic acid 0.1 ng/ml. Supplemented with penicillin/streptomycin, ofloxacin, and amphotericin B. | S5 |
| *Epomops buettikoferi* | Kidney | - | ✓ | SV40T | Tissue was lysed with 0.05% Trypsin and plated on 6-well tissue culture dishes in medium. | DMEM supplemented with 10% foetal calf serum and antibiotics (100 U/ml penicillin G and 100 μg/ml streptomycin) | S10 |
| *Epomophorus gambianus* | Kidney | - | ✓ | SV40 | - | MEM with 10% FCS, L-glutamine, and penicillin-streptomycin. | S11 |
| *Eptesicus fuscus* | Kidney | Epithelial/fibroblast | ✓ | Myotis polyomavirus T-antigen | Tissues were minced and incubated at room temperature in 0.5% trypsin-EDTA. Trypsin was neutralised with FBS. Cells were resuspended in medium, placed in 75 cm^2^ flasks and incubated at 37°C @ 5% CO_2_. | DMEM containing penicillin, streptomycin and amphotericin B, placed in 75 cm^2^ flasks | S12 |
| *Eptesicus nilssonii* | Kidney | - | ✓ | - | Tissues were minced and then digested with PBS containing 0.05% Trypsin and 0.025% EDTA at room temperature for 20 min, this solution was then mixed with DMEM/F12 containing 10% FCS and then centrifuged at 1000 g for 5 minutes, at which point debris was removed with a cell strainer. | DMEM/F12 or RPMI-1640, both supplemented with 10% FCS, NEAA, Sodium Pyruvate, Gentamycin, Penicillin, Streptomycin and Amphotericin B.  Then at passage 40 switched to DMEM with 10% FCS. | S13 |
|  | Lung | - | ✓ | - |  | DMEM/F12 or RPMI-1640, both supplemented with 10% FCS, NEAA, Sodium Pyruvate, Gentamycin, Penicillin, Streptomycin and Amphotericin B. |  |
|  | Spleen | - | ✓ | - |  | DMEM/F12 or RPMI-1640, both supplemented with 10% FCS, NEAA, Sodium Pyruvate, Gentamycin, Penicillin, Streptomycin and Amphotericin B. |  |
| *Hipposideros pomona* | Kidney | - | ✓ |  | Tissues were rinsed with cold PBS and minced. Cold 0.25% trypsin-EDTA was added to the tissues and this was incubated at 4°C overnight. Tissues were then incubated at 37°C on shaking platform for 30 min. Supernatants were filtered through cell strainers to remove large pieces of tissues. Bat cells were harvested by spinning down the supernatant at 1200 rpm for 8 min before plating. | DMEM/F12 supplemented with 15% FBS | S14 |
| *Hypsignathus monstrosus* | Kidney | - | ✓ | SV40T | Tissue was lysed with 0.05% Trypsin and plated on 6-well tissue culture dishes in medium. | DMEM supplemented with 10% foetal calf serum and antibiotics (100 U/ml penicillin G and 100 μg/ml streptomycin) | S10 |
|  | Lung | - | ✓ | SV40T |  |  |  |
| *Miniopterus fuliginosus* | Kidney | - | ✓ | SV40T | - | RPMI-1640 supplemented with 10% FBS | S11 |
| *Miniopterus pusillus* | Kidney | - | ✓ |  | Tissues were rinsed with cold PBS and minced. Cold 0.25% trypsin-EDTA was added to the tissues and this was incubated at 4°C overnight. Tissues were then incubated at 37°C on shaking platform for 30 min. Supernatants were filtered through cell strainers to remove large pieces of tissues. Bat cells were harvested by spinning down the supernatant at 1200 rpm for 8 min before plating. | DMEM/F12 supplemented with 15% FBS | S14 |
| *Miniopterus schreibersii* | Kidney | - | ✓ | SV40 | - | MEM with 10% FCS, L-glutamine, and penicillin-streptomycin. | S11 |
|  | Kidney | - | ✓ | - | Cold 0.25% Trypsin in PBS containing 200 mg/l disodium EDTA added to minced tissue and incubated at 4°C overnight. Tissues were then incubated at 37°C on a shaking platform for 1 h. Suspension then centrifuged @ 800 g for 5 min. | DMEM-F12-Hams supplemented with 10% foetal calf serum (FCS) and Antibiotic-Antimycotic antibiotics | S15 |
|  | Lymph node | - | ✓ | - |  |  |  |
| *Murina muricola* | Lung | Fibroblast | ✓ | Self-immortalised | Tissues were sliced into 1 mm sized pieces and incubated in a 0.22 µm filtered collagenase solution at 37°C with rotation (60 rpm) for 1 h to dissociate cells. Dissociated tissues were centrifuged at 430 g for 5 min, resuspended in medium and plated in T-25 culture flasks. Cells were maintained at 37°C with 5% CO_2_ until confluence. After the second passage, all cell lines were grown in medium. | Initial culture - RPMI medium 1640 (10% foetal bovine serum, 2% penicillin/streptomycin, 2.5%  After second passage - DMEM with 10% foetal bovine serum and penicillin/ streptomycin | S7 |
| *Myotis daubentonii* | Kidney | - | ✓ | SV40 | - | - | S8 |
|  | Lung | - | ✓ | SV40T | Tissue was lysed with 0.05% Trypsin and plated on 6-well tissue culture dishes in medium. | DMEM supplemented with 10% foetal calf serum and antibiotics (100 U/ml penicillin G and 100 μg/ml streptomycin) | S10 |
| *Myotis davidii* | Kidney | - | ✓ | Self-immortalised | Tissues were sliced into 1 mm sized pieces and incubated in a 0.22 µm filtered collagenase solution at 37°C with rotation (60 rpm) for 1 h to dissociate cells. Dissociated tissues were centrifuged at 430 g for 5 min, resuspended in medium and plated in T-25 culture flasks. Cells were maintained at 37°C with 5% CO_2_ until confluence. After the second passage, all cell lines were grown in medium. | Initial culture - RPMI medium 1640 (10% foetal bovine serum, 2% penicillin/streptomycin, 2.5%  After second passage - DMEM with 10% foetal bovine serum and penicillin/ streptomycin | S7 |
| *Myotis lucifugus* | Embryo | Fibroblast | *✓* | *-* | *-* | DMEM, 15% foetal bovine serum, 0.5% GlutaMax, 1.0% non-essential amino acid, 1 mM sodium pyruvate, and 0.5% penicillin and streptomycin. | S16 |
|  | Skin | Fibroblast | *✓* | *-* | 5mm diameter biopsies were minced into small pieces and then digested in Collagenase 2 overnight within growth media at 37°C 5% CO_2_. | DMEM, 20% FBS and antibiotics containing 100 U/ml penicillin, 100 μg/mL streptomycin and 0.25 μg/ml of fungizone. | S17 |
|  |  |  |  |  | - | DMEM and 10% Cosmic Calf Serum. | S18 |
| *Myotis myotis* | Brain | Fibroblast | ✓ | SV40T | Tissues were minced and cultured in 6-well plates until confluence reached 50–70%. | Dulbecco’s Modified Eagle Medium (DMEM) supplemented with 10% foetal calf serum (FCS), penicillin 100 units/mL and streptomycin 100 mg/mL (Sigma-Aldrich). | S19 |
|  | Tonsil | Fibroblast | ✓ | SV40T |  |  |  |
|  | Peritoneal cavity | Epithelial | ✓ | SV40T |  |  |  |
|  | Nasal epithelium | Fibroblast | ✓ | SV40T |  |  |  |
|  | Nervus olfactorius | Fibroblast | ✓ | SV40T |  |  |  |
|  | Wing | Fibroblast | ✓ | - | 3mm wing punches are cut into 0.5mm fragments, then resuspended in 0.2% Collagenase 2 growth medium in a 3 cm cell culture dish overnight at 37°C 5% CO_2_. Fresh growth medium is then applied after aspirating out the digestion mix but with a reduced 0.2% antibiotic concentration. | Dulbecco’s MEM high glucose with stabilised glutamine, 20% FBS and 1% antibiotics (Penicillin-Streptomycin-Fungizone), | S20 |
|  | Kidney | - | ✓ | - | Organs were washed in PBS containing 5% Antibiotic-Antimycotic mix and then transferred to a Eppendorf containing 1ml of 0.5% Trypsin diluted in DMEM and left for one 1 h in a shaker at 37°C. The cells were then transferred through a 100 μm cell strainer to a 10 cm cell culture plate with the strainer being washed through at medium flow. | DMEM + 10% Bovine Calf Serum supplemented with penicillin at 100 IU/mL and streptomycin at 100 μg/mL antibiotics. | S21 |
| *Myotis ricketti* | Kidney | - | - | - | Tissues were rinsed with cold PBS and minced. Cold 0.25% trypsin-EDTA was added to the tissues and this was incubated at 4°C overnight. Tissues were then incubated at 37°C on shaking platform for 30 min. Supernatants were filtered through cell strainers to remove large pieces of tissues. Bat cells were harvested by spinning down the supernatant at 1200 rpm for 8 min before plating. | DMEM/F12 supplemented with 15% FBS | S14 |
|  | Lung | - | - | - |  |  |  |
| *Nyctalus noctula* | Kidney | - | ✓ | - | Organs were washed in PBS containing 5% Antibiotic-Antimycotic mix and then transferred to a Eppendorf containing 1ml of 0.5% Trypsin diluted in DMEM and left for one 1 h in a shaker at 37°C. The cells were then transferred through a 100 μm cell strainer to a 10 cm cell culture plate with the strainer being washed through at medium flow. | DMEM + 10% Bovine Calf Serum supplemented with penicillin at 100 IU/mL and streptomycin at 100 μg/mL antibiotics. | S21 |
|  | Liver | - | ✓ | - |  |  |  |
| *Perimyotis subflavus* | Lung | Fibroblast | ✓ | hTERT and Bmi-1 | Tissues prepared according to Crameri et al. (2009). Organ tissues finely minced and rinsed with cold medium. Fragments were then transferred to a 50 ml conical tube, and cold 0.25% trypsin was added before overnight incubation at 4°C. Samples then shaken at 37°C on a benchtop shaker at 200 rpm for 1 h. Supernatants were filtered through a 40 μm cell strainer into a 50 ml conical tube containing 10 ml of foetal calf serum (FCS). Larger pieces of dissected tissues were incubated with 0.25% trypsin (Gibco) at 37°C for 30 min on the shaker, and then the supernatants added to FCS in the conical tube. Cells were pelleted at 800 × g for 5 min  Cells were then resuspended in medium, transferred to T25 flasks, and incubated at 37°C with 5% CO_2_. | DMEM/F12-Ham's media supplemented with 15% Foetal Clone II, 1% penicillin/streptomycin, 1% nonessential amino acids, and 1% gentamicin. | S22 |
|  | Heart | - | ✓ | - |  |  |  |
|  | Brain | - | ✓ | - |  |  |  |
|  | Kidney | - | ✓ | - |  |  |  |
| *Pipistrellus abramus* | Kidney | - | - | - | Tissues were rinsed with cold PBS and minced. Cold 0.25% trypsin-EDTA was added to the tissues and this was incubated at 4°C overnight. Tissues were then incubated at 37°C on shaking platform for 30 min. Supernatants were filtered through cell strainers to remove large pieces of tissues. Bat cells were harvested by spinning down the supernatant at 1200 rpm for 8 min before plating. | DMEM/F12 supplemented with 15% FBS | S14 |
|  | Lung | - | - | - |  |  |  |
| *Pipistrellus ceylonicus* | Embryonic | - | ✓ | - | Tissues washed with PBS, minced, and plated in 25 cm^2^ bottles with DMEM. Cells were supplemented with medium and maintained at 37°C, 5% CO_2_. When cells began attachment, dead cells were discarded and the medium was changed. After the third day, trypsinization was done and cells were passaged. | DMEM supplemented with 10% foetal bovine serum (FBS), 100 U penicillin/ml, and 0.1 mg streptomycin/ml | S23 |
| *Pipistrellus kuhlii* | Wing | Fibroblast | ✓ | - | 3mm wing punches are cut into 0.5mm fragments, then resuspended in 0.2% Collagenase 2 growth medium in a 3cm cell culture dish overnight at 37°C 5% CO_2_. Fresh growth medium is then applied after aspirating out the digestion mix but with a reduced 0.2% antibiotic concentration. | Dulbecco’s MEM high glucose with stabilised glutamine, 20% FBS and 1% antibiotics (Penicillin-Streptomycin-Fungizone), | S20 |
| *Pipistrellus nathusii* | Kidney | - | ✓ | - | Tissues were crushed and digested in 0.25% trypsin solution and 0.02% Versene solution three times using a magnetic stirrer at 37°C followed by cell straining. | Eagle's MEM and DMEM nutrient media supplemented with 10% FBS and 10 µg/ml of ciprofloxacin and 5 µg/ml of amphotericin. | S24 |
| *Pipistrellus pipistrellus* | Kidney | - | ✓ | SV40 | - | - | S4 |
|  | Kidney | - | ✓ | - | Organs were washed in PBS containing 5% Antibiotic-Antimycotic mix and then transferred to a Eppendorf containing 1ml of 0.5% Trypsin diluted in DMEM and left for one 1 h in a shaker at 37°C. The cells were then transferred through a 100 μm cell strainer to a 10 cm cell culture plate with the strainer being washed through at medium flow. | DMEM + 10% Bovine Calf Serum supplemented with penicillin at 100 IU/mL and streptomycin at 100 μg/mL antibiotics. | S21 |
| *Pteropus alecto* | Aorta | - | ✓ | SV40T/hTERT | Cold 0.25% Trypsin in PBS containing 200 mg/l disodium EDTA added to minced tissue and incubated at 4°C overnight. Tissues were then incubated at 37°C on a shaking platform for 1 h. Suspension then centrifuged @ 800 g for 5 min. | DMEM/F12-Hams, supplemented with 15% bovine calf serum (BCS, Hyclone), 100 units/ml penicillin, 100 µg/ml streptomycin and 50 µg/ml gentamycin (Sigma-Aldrich). | S25 |
|  | Bone Marrow | - | ✓ | SV40T |  |  |  |
|  | Brain | Neural | ✓ | SV40T/hTERT |  |  |  |
|  | Foetus | - | ✓ | SV40T/hTERT |  |  |  |
|  | Foetal membrane | - | ✓ | SV40T |  |  |  |
|  | Heart | - | ✓ | SV40T/hTERT |  |  |  |
|  | Kidney | Cuboidal | ✓ | SV40T/hTERT |  |  |  |
|  | Liver | - | ✓ | SV40T/hTERT |  |  |  |
|  | Lymph Nodes | - | ✓ | - |  |  |  |
|  | Lung | Cuboidal | ✓ | SV40T/hTERT |  |  |  |
|  | Muscle | - | ✓ | SV40T/hTERT |  |  |  |
|  | Pharynx | - | ✓ | - |  |  |  |
|  | Placenta | - | ✓ | SV40T/hTERT |  |  |  |
|  | Salivary Gland | - | ✓ | - |  |  |  |
|  | Small Intestine | - | ✓ | SV40T/hTERT |  |  |  |
|  | Skin | - | ✓ | - |  |  |  |
|  | Spleen | - | ✓ | SV40T/hTERT |  |  |  |
|  | Testes | - | ✓ | SV40T |  |  |  |
|  | Thymus | - | ✓ | - |  |  |  |
|  | Uterus | - | ✓ | SV40T/hTERT |  |  |  |
|  | Blood | PBMC’s | ✓ | - | Blood is added to ficoll-plaque PLUS and spun with the layer containing cells including RBC is lysed with RBC lysis buffer. | RPMI | S26 |
|  | Bone Marrow | - | ✓ | - | Flushed with RPMI, centrifuged, rinsed with RPMY and RBC lysed. |  |  |
|  | Brain | - | ✓ | - | Tissue was cut in 0.5 cm x 0.5 cm pieces and ground with a plunger within RPMI over a 100 μm filter, with subsequent centrifugation and wash steps. |  |  |
|  | Fat | - | ✓ | - | Tissue was cut in 0.5 cm x 0.5 cm pieces and ground with a plunger within RPMI over a 100 μm filter, with subsequent centrifugation and wash steps. |  |  |
|  | Heart | - | ✓ | - | Tissue was cut in 0.5 cm x 0.5 cm pieces and ground with a plunger within RPMI over a 100 μm filter, followed by digestion in a collagenase mix at 37°C for 10-30 min with subsequent centrifugation and wash steps. |  |  |
|  | Intestine (Large and small) | - | ✓ | - | Tissue was cut in 0.5 cm x 0.5 cm pieces and ground with a plunger within RPMI over a 100 μm filter, with subsequent centrifugation and wash steps. |  |  |
|  | Kidney | - | ✓ | - | Tissue was cut in 0.5 cm x 0.5 cm pieces and ground with a plunger within RPMI over a 100 μm filter, with subsequent centrifugation and wash steps. |  |  |
|  | Liver | - | ✓ | - | Tissue was cut in 0.5 cm x 0.5 cm pieces and ground with a plunger within RPMI over a 100 μm filter, with subsequent centrifugation and wash steps. |  |  |
|  | Lung | - | ✓ | - | Tissue was cut in 0.5 cm x 0.5 cm pieces and ground with a plunger within RPMI over a 100 μm filter, followed by digestion in a collagenase mix at 37°C for 10-30 min with subsequent centrifugation and wash steps. |  |  |
|  | Lymph Nodes | - | ✓ | - | Tissue was cut in 0.5 cm x 0.5 cm pieces and ground with a plunger within RPMI over a 100 μm filter, with subsequent centrifugation and wash steps. RBC lysation performed next. |  |  |
|  | Muscle | - | ✓ | - | Tissue was cut in 0.5 cm x 0.5 cm pieces and ground with a plunger within RPMI over a 100 μm filter, with subsequent centrifugation and wash steps. |  |  |
|  | Salivary Glands | - | ✓ | - | Tissue was cut in 0.5 cm x 0.5 cm pieces and ground with a plunger within RPMI over a 100 μm filter, with subsequent centrifugation and wash steps. |  |  |
|  | Skin | - | ✓ |  | Tissue was cut in 0.5 cm x 0.5 cm pieces and ground with a plunger within RPMI over a 100 μm filter, followed by digestion in a collagenase mix at 37°C for 10-30 min with subsequent centrifugation and wash steps. |  |  |
|  | Spleen | - | ✓ | - | Tissue was cut in 0.5 cm x 0.5 cm pieces and ground with a plunger within RPMI over a 100 μm filter, with subsequent centrifugation and wash steps. |  |  |
|  | Thymus | - | ✓ | - | Tissue was cut in 0.5 cm x 0.5 cm pieces and ground with a plunger within RPMI over a 100 μm filter, with subsequent centrifugation and wash steps. RBC lysation performed next. |  |  |
|  | Thyroids | - | ✓ | - | Tissue was cut in 0.5 cm x 0.5 cm pieces and ground with a plunger within RPMI over a 100 μm filter, with subsequent centrifugation and wash steps. |  |  |
| *Pteropus dasymallus* | Kidney | - | ✓ | SV40 | - | RPMI-1640 supplemented with 10% FBS | S11 |
|  | Kidney | - | ✓ | - | - | - | S27 |
|  | Spleen | - | ✓ | - | - | - |  |
| *Pteropus giganteus* | Spleen | - | ✓ | SV40 | - | RPMI-1640 supplemented with 10% FBS | S9 |
| *Pteropus pselaphon* | Finger | - | ✓ | CDK4, CYCLIN D1, and TERT | - | DMEM/F12 with 10% FBS and 1% Antibiotics | S28 |
|  | Wing | - | ✓ |  |  |  |  |
| *Rhinolophus ferrumequinum* | Kidney | - | ✓ | SV40T | Not Specified | RPMI-1640 supplemented with 10% FBS. | S27 |
| *Rhinolophus hipposideros* | Wing | Fibroblast | ✓ | - | Tissues minced and digested in a filter-sterilised 0.1% collagenase type II/DMEM solution for 12 h at 37°C, 5% CO_2_. Digestion solution was then removed with a pipette, and 2ml of culture medium was added. Plates were left undisturbed in the incubator for 1 week. When fibroblasts attachment/growth was visible, 1ml of medium was removed and replaced with DMEM supplemented with 0.2% Penicillin-Streptomycin and 20% Amniogrow plus (modified alpha-MEM with FCS, hormones, growth factors, L-glutamine and gentamycin). First passage of the culture was possible after ∼ 3 weeks. | DMEM supplemented with 1% of Penicillin-Streptomycin-Fungizone. | S29 |
| *Rhinolophus lepidus* | Kidney | - | - | Spontaneous | Tissue was mechanically pushed through a 100 µm cell strainer. Cells were then collected and plated at 100k in number onto a T25. In incubator set at 37°C and 5% CO_2._ | RPMI with L-Glutamine, 10% FBS, 2.5% HEPES and 1x antibiotics. | S30 |
|  | Large intestine | Fibroblast | ✓ | - | Tissues were sliced into 1 mm sized pieces and incubated in a 0.22 µm filtered collagenase solution at 37°C with rotation (60 rpm) for 1 h to dissociate cells. Dissociated tissues were centrifuged at 430 g for 5 min, resuspended in medium and plated in T-25 culture flasks. Cells were maintained at 37°C with 5% CO_2_ until confluence. After the second passage, all cell lines were grown in medium. | Initial culture - RPMI medium 1640 (10% foetal bovine serum, 2% penicillin/streptomycin, 2.5%  After second passage - DMEM with 10% foetal bovine serum and penicillin/ streptomycin | S7 |
| *Rhinolophus sinicus* | Kidney | - | ✓ | - | Tissues were rinsed with cold PBS and minced. Cold 0.25% trypsin-EDTA was added to the tissues and this was incubated at 4°C overnight. Tissues were then incubated at 37°C on shaking platform for 30 min. Supernatants were filtered through cell strainers to remove large pieces of tissues. Bat cells were harvested by spinning down the supernatant at 1200 rpm for 8 min before plating. | DMEM/F12 supplemented with 15% FBS | S14 |
|  | Lung | - | ✓ | - |  |  |  |
|  | Bladder | - | ✓ | - | Tissue was minced into pieces using sterile scissors and enzymatically digested in 5ml of 0.4 mg/ml collagenase IV, 0.4 mg/ml collagenase / dispase, 30 U/ml DNase, 0.5% BSA in HBSS, at 37°C, 100 rpm for 30 min. Following mechanical dissociation through repetitive pipetting cells were passed through a 70 nm strainer and cells were spun at 300 g for 5 min at 4°C and treated with red blood cell lysis agent. All the treated cells were finally diluted to a density of 1000 cells/μl in DPBS with 0.4% BSA. | - | S31 |
|  | Brain | - | ✓ | - |  |  |  |
|  | Brown + White Adipose Tissue | - | ✓ | - |  |  |  |
|  | Liver | - | ✓ | - |  |  |  |
|  | Lung | Fibroblast | ✓ | - |  | Lung fibroblasts were expanded further and this was done within RPMI 1640 medium with 10% foetal bovine serum and 1% penicillin (10,000 IU)-streptomycin (10,000 μg/mL) and then cultured in a 24-well culture plate for 48 h. The culture medium was replaced by Fibroblast Medium containing 2% FBS and 1% PS. |  |
|  | Pancreas | - | ✓ | - |  | - |  |
|  | Thymus | - | ✓ | - |  |  |  |
|  | Trachea | - | ✓ | - |  |  |  |
|  | Intestine | - | ✓ | - | Tissue was minced into pieces using sterile scissors and enzymatically digested in 5ml of 0.4 mg/ml collagenase II, 30 U/ml DNase, 0.5% BSA in HBSS, at 37°C, 100 rpm for 60 min. Following mechanical dissociation through repetitive pipetting cells were passed through a 70 nm strainer and cells were spun at 300 g for 5 min at 4°C and treated with red blood cell lysis agent. All the treated cells were finally diluted to a density of 1000 cells/μl in DPBS with 0.4% BSA. |  |  |
|  | Muscle | - | ✓ | - |  |  |  |
|  | Testis | - | ✓ | - |  |  |  |
|  | Heart | - | ✓ | - | Tissue was minced into pieces using sterile scissors and enzymatically digested in 5 ml of 1 mg/ml collagenase/dispase, 30 U/ml DNase, 0.5% BSA in HBSS, at 37°C, 100 rpm for 45 min. Following mechanical dissociation through repetitive pipetting cells were passed through a 70 nm strainer and cells were spun at 300 g for 5 min at 4°C and treated with red blood cell lysis agent. All the treated cells were finally diluted to a density of 1000 cells/μl in DPBS with 0.4% BSA. |  |  |
|  | Kidney | - | ✓ | - | Tissue was minced into pieces using sterile scissors and enzymatically digested in 5ml of 0.25% Trypsin and 30 U/ml DNase in 0.5% BSA in HBSS, at 37°C for 15 min. Following mechanical dissociation through repetitive pipetting cells were passed through a 70 nm strainer and cells were spun at 300 g for 5 min at 4°C and treated with red blood cell lysis agent. All the treated cells were finally diluted to a density of 1000 cells/μl in DPBS with 0.4% BSA. |  |  |
|  | Wing | - | ✓ | - | Tissue was minced into pieces using sterile scissors and enzymatically digested in 5ml of 0.25% Trypsin and 30 U/ml DNase, in 0.5% BSA in HBSS at 37°C for 30 min. Following mechanical dissociation through repetitive pipetting cells were passed through a 70 nm strainer and cells were spun at 300 g for 5 min at 4°C and treated with red blood cell lysis agent. All the treated cells were finally diluted to a density of 1000 cells/μl in DPBS with 0.4% BSA. |  |  |
|  | Tongue | - | ✓ | - | Tissue was minced into pieces using sterile scissors and enzymatically digested in 5ml of 0.4 mg/ml collagenase IV, 30 U/ml DNase, 0.5% BSA in HBSS, at 37°C, 100 rpm for 60 min. Following mechanical dissociation through repetitive pipetting cells were passed through a 70nm strainer and cells were spun at 300 g for 5 min at 4°C and treated with red blood cell lysis agent. All the treated cells were finally diluted to a density of 1000 cells/μl in DPBS with 0.4% BSA. |  |  |
|  | Blood | - | ✓ | - | Blood was added to EDTA and mixed followed by suspension with 0.5 ml of red blood cell lysis buffer. The cell suspension was incubated on ice for 1 minute and the lysis reaction was quenched by adding 10 ml Dulbecco’s Phosphate Buffered Saline with 2 mM EDTA and 0.5% BSA. Cells were then collected at 200g for 5 min at 4°C and washed with DPBS twice. |  |  |
|  | Bone Marrow | - | ✓ | - | The bone marrows were flushed with DPBS with the liquid flush being applied through a 70 μm cell strainer. Cells were centrifuged at 200 g for 5 min at 4°C before being resuspended in 3 ml of red blood cell lysis buffer on ice for 1 minute with the lysis reaction being quenched with 20 ml DPBS with 2 mM EDTA and 0.5% BSA. With a final collection of cells for use through a 300g centrifuge spin for five min at 4°C. |  |  |
|  | Spleen | - | ✓ | - | Spleens were rinsed in cold DPBS, then using a plunger were squeezed through a 70 μm cell strainer using plungers. The cells were then centrifuged at 300 g for five min at 4°C before being resuspended in 3 ml of red blood cell lysis buffer on ice for 1 min with the lysis reaction being quenched with 20 ml DPBS with 2 mM EDTA and 0.5% BSA. With a final collection of cells for use through a 300 g centrifuge spin for five min at 4°C. |  |  |
| *Rousettus aegyptiacus* | Kidney | - | ✓ | SV40T | Tissue was lysed with 0.05% Trypsin and plated on 6-well tissue culture dishes in medium. | DMEM supplemented with 10% foetal calf serum and antibiotics (100 U/ml penicillin G and 100 μg/ml streptomycin) | S10 |
|  | Kidney | - | ✓ | SV40 | - | - | S8 |
|  | Embryonic | - | ✓ | Transfected with an expression plasmid for adenovirus serotype 5 E1A and E1B open reading frames. | Amniotic fluid, cells from brain, liver, and the vertebrate column were carefully removed and transferred separately into culture medium. Remaining body segments were treated with TrypLE (Gibco) before being placed into culture medium. Cells from a total of 14 preparations were seeded into 6-well culture plates and maintained at 37°C and 8% CO_2_. | *-* | S32 |
| *Rousettus leschenaultii* | Kidney | - | ✓ | SV40 | - | RPMI-1640 supplemented with 10% FBS | S11 |
|  | Kidney | - | ✓ | - | Tissues were rinsed with cold PBS and minced. Cold 0.25% trypsin-EDTA was added to the tissues and this was incubated at 4°C overnight. Tissues were then incubated at 37°C on shaking platform for 30 min. Supernatants were filtered through cell strainers to remove large pieces of tissues. Bat cells were harvested by spinning down the supernatant at 1200 rpm for 8 min before plating. | DMEM/F12 supplemented with 15% FBS | S14 |
|  | Lung | - | ✓ | - |  |  |  |
|  | Intestines | Intestinal Crypt Cells / Organoids | ✓ | - | Samples were washed with PBS cut into small pieces and digested with 0.125mg/m at 37°C for 30 min with pipetting of media every 15 min. Cells were then passed through a 70 µm nylon net cell strainer. | DMEM | S33 |
| *Tadarida brasiliensis* | Lung | Epithelial | ✓ | - | - | - | S34 |
| *Tylonycteris pachypus* | Kidney | - | ✓ | - | Tissues were rinsed with cold PBS and minced. Cold 0.25% trypsin-EDTA was added to the tissues and this was incubated at 4°C overnight. Tissues were then incubated at 37°C on shaking platform for 30 min. Supernatants were filtered through cell strainers to remove large pieces of tissues. Bat cells were harvested by spinning down the supernatant at 1200 rpm for 8 min before plating. | DMEM/F12 supplemented with 15% FBS | S14 |
|  | Lung | - | ✓ | - |  |  |  |
| *Vespertilio murinus* | Kidney | - | ✓ | - | Organs were washed in PBS containing 5% Antibiotic-Antimycotic mix and then transferred to a Eppendorf containing 1ml of 0.5% Trypsin diluted in DMEM and left for one 1 h in a shaker at 37°C. The cells were then transferred through a 100 μm cell strainer to a 10 cm cell culture plate with the strainer being washed through at medium flow. | DMEM + 10% Bovine Calf Serum supplemented with penicillin at 100 IU/mL and streptomycin at 100 μg/mL antibiotics. | S21 |

**Supplemental Table 2:** Results of digestion experiment comparing digestion method and post growth media condition effects across three individuals with 3 biological repeats (individual wing punches) per bat per condition. Cell numbers counted manually are shown with their averages. The bat with designation R49 had one cell culture failure due to a contamination and was thus counted as not having expanded successfully.

| **Cell Culture Reference Code** | **DMEM +**  **Collagenase** | | **DMEM +**  **Collagenase + Elastase** | | **FGM +**  **Collagenase + Elastase** | |  |  |
| --- | --- | --- | --- | --- | --- | --- | --- | --- |
|  | **Cell Count for each bi logical repeat** | **Average Cell Count** | **Cell Count for each biological repeat** | **Average Cell Count** | **Cell Count for each biological repeat** | **Average Cell Count** |  |  |
| ***R49*** | R1:  185,000 | 143,333 | R1:  140,000 | 61,667 | R1:  435,000 | 530,000 |  |  |
|  | R2:  122,500 |  | R2:  45,000 |  | R2:  512,500 |  |  |  |
|  | R3:  122,500 |  | R3:  0 |  | R3:  642,500 |  |  |  |
| ***R51*** | R1:  58,500 | 135,917 | R1:  141,250 | 75,000 | R1:  93,750 | 249,583 |  |  |
|  | R2:  123,000 |  | R2:  67,500 |  | R2:  166,250 |  |  |  |
|  | R3:  226,250 |  | R3:  16,250 |  | R3:  488,750 |  |  |  |
| ***R56*** | R1:  64,000 | 67,000 | R1:  17,000 | 28,000 | R1:  70,000 | 451,250 |  |  |
|  | R2:  40,000 |  | R2:  32,000 |  | R2:  706,250 |  |  |  |
|  | R3:  97,000 |  | R3:  35,000 |  | R3:  577,500 |  |  |  |

**Supplemental Table 3:** Blast results of Species Identification conducted through Cytochrome b detection primers.

| Culture Name | Gene | Species | Max Score | Total Score | Query Cover | E value | Percentage Identity | Accension Length | Accession Number |
| --- | --- | --- | --- | --- | --- | --- | --- | --- | --- |
| R42 | Cytb | *R,aegyptiacus* | 1290 | 1290 | 100% | 0.0 | 99.86% | 864 | MH999931.1 |
| R42IM | Cytb | *R,aegyptiacus* | 1391 | 1391 | 100% | 0.0 | 99.22% | 867 | JX274466.1 |
| R46 | Cytb | *R,aegyptiacus* | 1386 | 1386 | 100% | 0.0 | 100% | 867 | JX274479.1 |
| R46IM | Cytb | *R,aegyptiacus* | 1437 | 1437 | 100% | 0.0 | 99.75% | 16706 | NC_007393.1 |
| R49 | Cytb | *R,aegyptiacus* | 1391 | 1391 | 100% | 0.0 | 100% | 16753 | MN816349.1 |
| R49IM | Cytb | *R,aegyptiacus* | 1421 | 1421 | 100% | 0.0 | 99.36% | 16753 | MN816349.1 |

**Supplemental References:**

[S1]. Porras-Gómez, T. J. & Moreno-Mendoza, N. Interaction between oocytes, cortical germ cells and granulosa cells of the mouse and bat, following the dissociation–re-aggregation of adult ovaries. *Zygote* **28**, 223–232 (2020).

[S2]. Carvalho, V. S., Rissino, J. D., Nagamachi, C. Y., Pieczarka, J. C. & Noronha, R. C. R. Isolation and establishment of skin-derived and mesenchymal cells from south American bat Artibeus planirostris (Chiroptera – Phyllostomidae). *Tissue Cell* **71**, 101507 (2021).

[S3]. Moratelli, R., Andrade, C. de M. & de Armada, J. L. A. A technique to obtain fibroblast cells from skin biopsies of living bats (Chiroptera) for cytogenetic studies. *Genet. Mol. Res. GMR* **1**, 128–130 (2002).

[S4]. Müller, M. A. *et al.* Human Coronavirus EMC Does Not Require the SARS-Coronavirus Receptor and Maintains Broad Replicative Capability in Mammalian Cell Lines. *mBio* **3**, e00515-12 (2012).

[S5]. Eckerle, I. *et al.* Bat Airway Epithelial Cells: A Novel Tool for the Study of Zoonotic Viruses. *PLoS ONE* **9**, e84679 (2014).

[S6]. Su, A. *et al.* Infection Studies with Airway Organoids from Carollia perspicillata Indicate That the Respiratory Epithelium Is Not a Barrier for Interspecies Transmission of Influenza Viruses. *Microbiol. Spectr.* **11**, e03098-22 (2023).

[S7]. Koh, J. *et al.* ABCB1 protects bat cells from DNA damage induced by genotoxic compounds. *Nat. Commun.* **10**, 2820 (2019).

[S8]. Biesold, S. E. *et al.* Type I Interferon Reaction to Viral Infection in Interferon-Competent, Immortalized Cell Lines from the African Fruit Bat Eidolon helvum. *PLOS ONE* **6**, e28131 (2011).

[S9]. Maruyama, J. *et al.* Characterization of the glycoproteins of bat-derived influenza viruses. *Virology* **488**, 43–50 (2016).

[S10]. Kühl, A. *et al.* Comparative Analysis of Ebola Virus Glycoprotein Interactions With Human and Bat Cells. *J. Infect. Dis.* **204**, S840–S849 (2011).

[S11]. Maruyama, J. *et al.* Characterization of the Envelope Glycoprotein of a Novel Filovirus, Lloviu Virus. *J. Virol.* **88**, 99–109 (2014).

[S12]. Banerjee, A. *et al.* Generation and Characterization of Eptesicus fuscus (Big brown bat) kidney cell lines immortalized using the Myotis polyomavirus large T-antigen. *J. Virol. Methods* **237**, 166–173 (2016).

[S13]. Horie, M., Akasaka, T., Matsuda, S., Ogawa, H. & Imai, K. Establishment and characterization of a cell line derived from Eptesicus nilssonii. *J. Vet. Med. Sci.* **78**, 1727–1729 (2016).

[S14]. Lau, S. K. P. *et al.* Replication of MERS and SARS coronaviruses in bat cells offers insights to their ancestral origins. *Emerg. Microbes Infect.* **7**, 209–209 (2018).

[S15]. Zhang, H. *et al.* A novel bat herpesvirus encodes homologues of major histocompatibility complex classes I and II, C-type lectin, and a unique family of immune-related genes. *J. Virol.* **86**, 8014–8030 (2012).

[S16]. Mo, X., Li, N. & Wu, S. Generation and characterization of bat-induced pluripotent stem cells. *Theriogenology* **82**, 283–293 (2014).

[S17]. Harper, J. M., Salmon, A. B., Leiser, S. F., Galecki, A. T. & Miller, R. A. Skin-derived fibroblasts from long-lived species are resistant to some, but not all, lethal stresses and to the mitochondrial inhibitor rotenone. *Aging Cell* **6**, 1–13 (2007).

[S18]. Pride, H. *et al.* Long-lived species have improved proteostasis compared to phylogenetically-related shorter-lived species. *Biochem. Biophys. Res. Commun.* **457**, 669–675 (2015).

[S19]. He, X. *et al.* Establishment of Myotis myotis Cell Lines - Model for Investigation of Host-Pathogen Interaction in a Natural Host for Emerging Viruses. *PLoS ONE* **9**, e109795 (2014).

[S20]. Kacprzyk, J. *et al.* Evolution of mammalian longevity: age-related increase in autophagy in bats compared to other mammals. *Aging* **13**, 7998–8025 (2021).

[S21]. Nemcova, M. *et al.* Bat-derived cells use glucose as a cryoprotectant. *J. Therm. Biol.* **115**, 103652 (2023).

[S22]. Huynh, J. *et al.* Evidence Supporting a Zoonotic Origin of Human Coronavirus Strain NL63. *J. Virol.* **86**, 12816–12825 (2012).

[S23]. Mourya, D. T. *et al.* Establishment of cell line from embryonic tissue of Pipistrellus ceylonicus bat species from India & its susceptibility to different viruses. *Indian J. Med. Res.* **138**, 224–231 (2013).

[S24]. Povolyaeva, O. S. *et al.* Biological characteristics and permissivity to viruses of a strain of diploid cells of the kidney of the bat Nathusius ( Pipistrellus nathusii )Keyserling & Blasius, 1839; ( Chiroptera: Microchiroptera: Vespertilionidae ). *Quest. Virol.* **66**, 29–39 (2021).

[S25]. Crameri, G. *et al.* Establishment, Immortalisation and Characterisation of Pteropid Bat Cell Lines. *PLoS ONE* **4**, e8266 (2009).

[S26]. Irving, A. T. *et al.* Optimizing dissection, sample collection and cell isolation protocols for frugivorous bats. *Methods Ecol. Evol.* **11**, 150–158 (2020).

[S27]. Maeda, K. *et al.* Isolation of Novel Adenovirus from Fruit Bat (Pteropus dasymallus yayeyamae). *Emerg. Infect. Dis. J.* **14**, 347 (2008).

[S28]. Tani, T. *et al.* Establishment of immortalized primary cell from the critically endangered Bonin flying fox (Pteropus pselaphon). *PloS One* **14**, e0221364 (2019).

[S29]. Kacprzyk, J., Teeling, E. C., Kelleher, C. & Volleth, M. Wing Membrane Biopsies for Bat Cytogenetics: Finding of 2n = 54 in Irish Rhinolophushipposideros (Rhinolophidae, Chiroptera, Mammalia) Supports Two Geographically Separated Chromosomal Variants in Europe. *Cytogenet. Genome Res.* **148**, 279–283 (2016).

[S30]. Mah, M. G. *et al.* Spike-Independent Infection of Human Coronavirus 229E in Bat Cells. *Microbiol. Spectr.* **11**, e03483-22 (2023).

[S31]. Ren, L. *et al.* Single-cell transcriptional atlas of the Chinese horseshoe bat (Rhinolophus sinicus) provides insight into the cellular mechanisms which enable bats to be viral reservoirs. 2020.06.30.175778 Preprint at https://doi.org/10.1101/2020.06.30.175778 (2020).

[S32]. Jordan, I., Horn, D., Oehmke, S., Leendertz, F. H. & Sandig, V. Cell lines from the Egyptian fruit bat are permissive for modiﬁed vaccinia Ankara. *Virus Res.* 10 (2009).

[S33]. Elbadawy, M. *et al.* Establishment of Intestinal Organoid from Rousettus leschenaultii and the Susceptibility to Bat-Associated Viruses, SARS-CoV-2 and Pteropine Orthoreovirus. *Int. J. Mol. Sci.* **22**, 10763 (2021).

[S34]. Stulberg, C. S., Coriell, L. L., Kniazeff, A. J. & Shannon, J. E. The animal cell culture collection. *In Vitro* **5**, 1–16 (1970).
